# Genomic Analysis of Latvian Brown Old Type and Latvian Blue Local Dairy Cattle Breeds Using SNP Data

**DOI:** 10.3390/ani16010020

**Published:** 2025-12-20

**Authors:** Daina Jonkus, Lasma Cielava, Didzis Dreimanis, Viktorija Nikonova, Liga Paura

**Affiliations:** Institute of Animal Sciences, Latvia University of Life Sciences and Technologies, Liela Street 2, LV 3001 Jelgava, Latvia; daina.jonkus@lbtu.lv (D.J.); lasma.cielava@lbtu.lv (L.C.); didzis.dreimanis@lbtu.lv (D.D.); viktorija.nikonova@lbtu.lv (V.N.)

**Keywords:** SNP data, inbreeding, heterozygosity, ROH, milk yield

## Abstract

Conservation programmes for two local dairy cattle breeds—Latvian Brown old type (BV) and Latvian Blue (LZ)—commenced in 2004. The aim of this study was to evaluate genetic diversity in the BV and LZ local cattle populations using SNP data. This study was based on genotype data from 96 BV and 75 LZ cows and 20 BV and 18 LZ bulls. The SNPs were determined using the GGP 100K bovine SNP BeadChip. Quality control (QC) and genotype data analysis were performed using PLINK v1.9. The observed heterozygosity was moderate, at around 0.4, for both breeds. Inbreeding coefficients were estimated based on homozygosity runs (F_ROH_) to compare recent and ancient inbreeding in the BV and LZ populations. Therefore, the ROH segments were divided into segments with the four classes (1–4 Mb, 4–8 Mb, 8–16 Mb, and above 16 Mb). Shorter ROH regions (ROH < 4 Mb) predominated in the genome. ROH regions with lengths above 16 Mb covers 4–6% of the genome in BV and 11% in LZ population. The average inbreeding coefficient for approximately three generations (F_ROH>16_) was 2.30% and 4.87% for BV and LZ cows (*p* < 0.05), respectively, and 2.59% and 3.85% for BV and LZ bulls, respectively. This study demonstrates that inbreeding has increased from generation to generation (F_ROH>16_ is higher compared with F_ROH<16_) in both populations. The level of current inbreeding in LZ is higher compared with that in the BV breed. The overall level of inbreeding in the BV and LZ populations is low, but there is a high level of inbreeding among a few animals. The impact of inbreeding on cow productivity has been observed in the LZ and BV cow populations. As a result, breeding organisations need to monitor and control the level of inbreeding and prevent the loss of genetic diversity in these animal populations. Breeders should minimize mating among close relatives; introduce genetically unrelated animals, use pedigree, and genomic information in controlling rates of inbreeding.

## Simple Summary

In Latvia, there is only a small number of the native dairy cattle breeds: Latvian Brown old type and Latvian Blue. The protection and breeding of these breeds, which are genetically adapted to traditional production systems, are very important. Using genome-wide SNP data, breed diversity and genetic variation were analysed. Recent and ancient inbreeding in the BV and LZ populations were estimated based on homozygosity runs. Regardless of the lower number of bulls, the Latvian Blue breed had a much higher level of inbreeding than the Latvian Brown old type. The relationship between genomic inbreeding and milk productivity and a negative phenotypic correlation between genomic inbreeding and milk yield for both breeds was observed. The Latvian local cow breeds are not characterised by high milk yields, but they do have a high dry milk matter content.

## 1. Introduction

In Latvia, two native dairy cattle breeds—Latvian Brown old type (BV) and Latvian Blue (LZ)—are officially recognised and protected as endangered genetic resources. Both breeds have been under active conservation since 2004 through national breeding programmes, coordinated by the Latvian Ministry of Agriculture.

The protection and breeding of these breeds are regulated under Regulation (EU) 2016/1012 [1], which defines an endangered breed as a local breed formally recognised by a Member State as being at risk of extinction, genetically adapted to traditional production systems, and scientifically validated by competent authorities. Conservation and breeding strategies for these breeds must be implemented by officially recognised animal breeding organisations. In Latvia, these responsibilities are taken on by the “Animal Breeders Association of Latvia” and the breed’s conservation association “The Blue Cow” [2], which develops and administers dedicated conservation and breeding plans for both local endangered breeds, which are included in the Domestic Animal Diversity Information System (DAD-IS) [3,4].

The Latvian Brown breed originates from native Baltic cattle, historically characterised by a small-sized body, hardiness, and adaptability to low-input systems. Selective improvement began in the mid-19th century through controlled mating with Angler (from 1862) and Danish Red (early 20th century). The Latvian Brown (LB) breed was officially recognised in 1922. During the latter half of the 20th century, these animals were mated with bulls of red and red-and-white breeds to achieve a higher level of milk productivity, but this did lower the amount of milk fat and protein, cattle sturdiness, and reproductive performance.

In 2004, a decision was made to initiate the preservation of the LB. As a result, animals with at least 50% LB ancestry were separated from the overall population. Animals with a documented four-generation pedigree, with at least 50% LB ancestry and purebred Danish Red and Angler in the pedigree, were accepted as genetic resources and defined as LB old type [5]. Starting from 2024, a new abbreviation for LB old type was accepted as BV. The BV breed is registered in a herd book which is separate from the LB new type.

The LZ breed was first registered in the 1930s, distinguished by its unique blue-grey coat coloration and adaptation to coastal and lowland farming systems. During the twentieth century, the breed experienced a severe population decline and extensive genetic dilution due to crossbreeding with commercial dairy breeds. Recovery efforts in the late 20th and early 21st centuries relied on a small number of surviving individuals, supplemented by controlled introgression from Tyrol Grey and Lithuanian Light Grey cattle to mitigate inbreeding depression while maintaining phenotypic distinctiveness. Animals classified as LZ must exhibit characteristic blue-grey pigmentation and demonstrate documented genetic continuity with the traditional Latvian Blue lineage [6].

**Figure 1:**
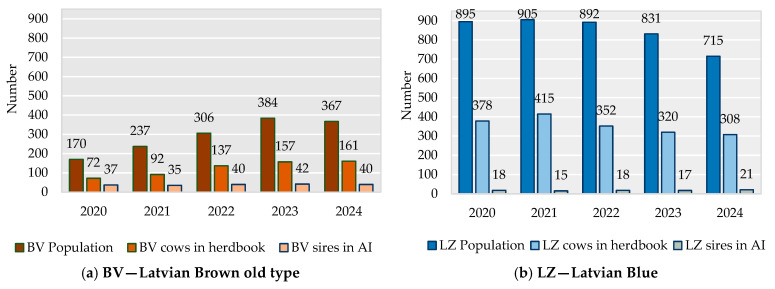
Size of BV (**a**) and LZ (**b**) populations; number of LZ and BV cows and bulls [6].

Over the last decade, the number of genetic resources of BV animals has increased, but that of LZ animals has decreased (Figure 1); however, the LZ population is larger than the BV population. This decrease in the LZ population can be explained by low cow milk productivity in comparison to the commercial breeds and the substitution of local cows with the Holstein breed. The effective population size (Ne) of the BV population over the last five years ranged from 98.7 animals in 2020 to 128.2 in 2024; that of LZ animals was lower, from 68.7 to 78.6 animals, during the analysed time period.

BV cows are kept on a total of 26 farms, of which 3 have more than 30 cows. Typically, these farms have between 5 and 10 cows. LZ herds are also small, with 10–20 dairy cows, and many cows are kept in herds of only 1–5 animals. In total, more than 40 farms are officially registered as keeping blue cows.

In Latvia, local breed cows are artificially inseminated. The breeding bioproduct is stored in three artificial insemination stations, where the bioproduct of 46 BV bulls and 24 LZ bulls is preserved.

Genetic diversity is important in animal populations, especially populations of local breeds, because their population sizes are often small. Preserving genetic diversity ensures that unique breed traits are not lost and provides a strong genetic base for future breeding and improvement programmes [7,8].

Several parameters can be used to measure overall genetic variation in the population. Observed and expected heterozygosity, coefficient of inbreeding, and effective population size are commonly used parameters in genetic diversity analysis of local populations [9,10,11,12,13].

Only a few studies on the diversity of Latvian cow breeds have been performed, and the genetic diversity of BV and LZ cattle has not been explored extensively. In the few studies that exist, Paura and Jonkus used pedigree data to assess the level of inbreeding for local Latvian Brown and Latvian Blue cattle breeds [14], and Gudra et al. performed the genetic characterisation of local Latvian dairy cattle breeds [15].

Similar to the study by Gudra et al. [15], our research focuses on Latvian cattle breeds. The current study provides additional information as we analyse inbreeding levels using the total length of ROH and compare recent versus ancient inbreeding in the BV and LZ populations based on data samples collected during our project. Furthermore, we present a preliminary evaluation of the impact of inbreeding on milk productivity traits, analyses that were not included in Gudra et al.’s study [15]. It is essential to perform regular evaluation and analysis of the genetic diversity within the genetic resource populations of Latvian animals. Therefore, the aim of this study is to (1) assess the genetic diversity of the Latvian Brown old type (BV) and Latvian Blue (LZ) local cattle populations using SNP genotype data; and (2) evaluate BV and LZ cows’ productivity, as well as the effect of inbreeding on cows’ productivity.

## 2. Materials and Methods

### 2.1. Animals’ Data

For DNA extraction and further molecular genetic analyses, hair samples from cow tails were collected from 96 BV and 75 LZ breed genetic resources cows (sampling locations for BV and LZ cows are in Figure S1). The samples were obtained during the cows’ health inspections, which were carried out by a certified person. Samples were collected in June–September of 2023 and 2024. Samples were taken from BV breed cows in three different herds: 68 samples were collected from the MPS “Vecauce” university teaching and research farm, and 21 and 7 cow samples were obtained from the other two farms. LZ breed cows were located on 15 traditional farms with a small number of animals (an average of five–ten cows per farm). All cows were born between 2018 and 2023. Samples were selected from animals registered in the BV and LZ breed herd book proportionally to the herd size to obtain a representative set from the active population.

Bull semen samples (AI-deposited doses) representing 20 BV and 18 LZ breed bulls were obtained from the artificial insemination station (AIS). All bulls were born after 2000, and only those with an appropriate number of doses at the AIS were included in the analysis.

### 2.2. SNP Typing and Quality Control

Samples from 171 cows and 38 bulls were genotyped. SNPs were determined using the GGP 100K bovine SNP BeadChip provided by Neogen Europe Limited (Ayr, Scotland, UK).

Quality control (QC) of the genotype data was performed using PLINK v1.9 [16,17] and to remove poorly genotyped markers and samples was applied missingness filter and an individual-level missingness filter of 10% (--geno 0.1 --mind 0.1) [18].

SNPs that did not meet these quality criteria were excluded from the analysis. In the cows’ dataset, a total of 83,628 SNPs and 169 cows passed the QC procedure; 1407 variants were removed due to missing genotype data.

In the bull dataset, a total of 83,601 SNPs and 38 bulls passed the QC procedure; 1255 variants were removed due to missing genotype data.

### 2.3. Between-Breed and Within-Breed Variation Analysis

After performing input data QC, principal component analysis (PCA) was used to visualise the genetic clustering of animals and the variability between and within populations. Principal components (PCs) of the genotype data were calculated using PLINK v1.9 via the --*pca* function. The PCs were plotted using R 4.5.0 (Vienna, Austria, R Core Team) [19].

### 2.4. ROH and Genomic Inbreeding Evaluation

Runs of homozygosity (ROH) for each animal were calculated using PLINK v1.9 via the --*homozyg* function. The parameters of the PLINK function were adjusted to the density of the SNP panel (GGP bovine 100K). An ROH was defined as a continuous stretch of at least 40 consecutive homozygous SNPs with a minimum length of 1 Mb. Within an ROH, one heterozygous and up to five missing genotypes were allowed. The maximum gap between two consecutive homozygous SNPs was defined to be 500 kb, and the minimum SNP density was one SNP per 75 kb. For the sliding window procedure, windows included 40 SNPs, allowing for one heterozygous genotype and up to five missing genotypes per window [18,20]. Windows with these criteria were classified as homozygous. The selected ROH parameters and quality control thresholds were chosen in accordance with controlling the type I error rate.

Inbreeding coefficients were estimated based on homozygosity runs F_ROH_  = * L_ROH_*/*L_aut_*, where *L_ROH_* is the total length of all ROHs in the animal’s genome and *L_aut_* is the length of the autosomal genome [21]. Inbreeding was calculated using the total ROH length and in four ROH length categories. To compare recent and ancient inbreeding in the BV and LZ populations, the ROH values were classified into four categories based on the ROH length: 1–4 Mb, 4–8 Mb, 8–16 Mb, and above 16 Mb [20,22,23]. The last group with ROH length above 16 Mb presents inbreeding around three generations, while long ROH indicates recent inbreeding, and short ROH presents ancient inbreeding [24].

The differences in inbreeding levels between the BV and LZ populations and between generations were tested using the Mann–Whitney U test.

### 2.5. Effect of Inbreeding on Cows’ Productivity

The 305-day productivity of the first lactation cows was used to analyse the effect of F_ROH_ inbreeding on cow milk yield. Productivity data from 78 BV and 48 LZ breed cows were obtained from the Agricultural Data Centre and included in the analysis. Individual inbreeding (F_ROH_) calculated from the total ROH length was used in this case.

## 3. Results

### 3.1. Genetic Variability and Clustering of Latvian Dairy Breeds

**Figure 2:**
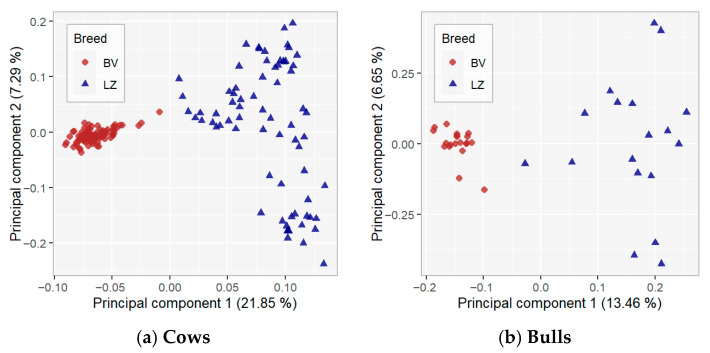
Principal component analysis results.

Principal component analysis (PCA) was used to visualise the genetic clustering of the animals and the variability between and within populations. The results of the PCA are presented in Figure 2, where the first principal component (PC1) explains 21.85% of the total variation, and PC2 explains 7.29%. The two principal components (PC1 and PC2) explained 29.14% of the total variation. The location of cows in the PC plot revealed two strong breed-specific clusters according to PC1. The variation among the LZ breed is higher compared with the BV breed. Results for the PC1–PC3 and PC2–PC3 analyses (Figure S2) indicate that the clustering patterns are stable across different component combinations, with breed separation remaining stable across orthogonal PCA dimensions.

### 3.2. Heterozygosity and Minor Allele Frequency

Observed heterozygosity (Ho) and minor allele frequency (MAF) describe the level of diversity in the BV and LZ populations (Table 1).

The MAF was relatively low, at around 0.32. The average Ho was similar between the two breeds in the cow and bull groups (Table 1). The observed heterozygosity was slightly higher for LZ cows and bulls than it was for the BV breed. The lowest proportion of heterozygous genotypes was in the BV genome (0.401 for cows and 0.397 for bulls).

### 3.3. Runs of Homozygosity (ROH)

**Figure 3:**
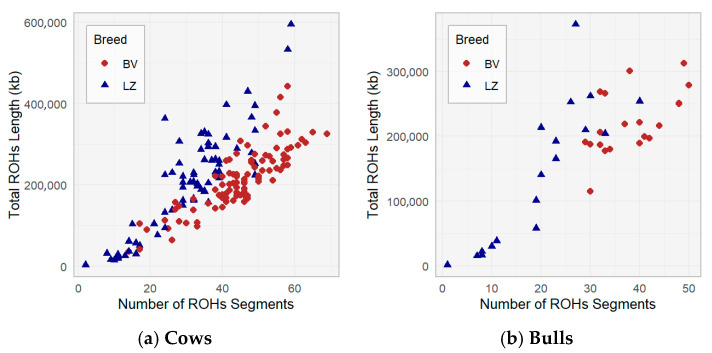
Number of ROH segments and total ROH length in the BV and LZ populations.

A comparison of the breeds in terms of their total ROH lengths and number of ROH is shown in Figure 3. A positive relationship was found among the number of ROH segments and the total ROH length in both populations: as the number of ROH segments increases, the total length of the ROH also increases; cows with a higher number of ROH segments typically have a longer total ROH (Figure 3a). The ROH length of some LZ population cows shows longer total ROH lengths, especially at higher segment numbers, compared with the BV breed. This suggests that LZ cows have a wider range of autozygosity than BV cows, and differences were observed related to breeding history and selection pressures in the BV population.

There is a difference between the BV (mean = 220,664 and SD = 48,399) and LZ (mean = 141,770 and SD = 110,341) bulls in terms of total ROH length (*p* < 0.05). BV bulls have longer total ROH lengths compared with LZ bulls (Figure 3b). These observed differences can be explained because semen doses are presented from closely related bulls, which presented four genetic lines in the BV population.

The statistics for the individual ROH segments category for BV and LZ cow and bull breeds are presented in Table 2 (Tables S1 and S2). The proportions of the genome in ROH were calculated for ROH 1–4 Mb, 4–8 Mb, 8–16 Mb, and above 16 Mb. In the cow and bull populations, shorter regions (1–4 Mb) predominated in the genome, representing 63% of ROH in BV and 60% in LZ in the cow data and 54% and 50% in the BV and LZ bull data, respectively. In the 4 to 8 Mb and 8 to 16 Mb ROHs, more than 29% of ROHs are present in the cow data, and more than 39% are present in the bull data. ROH lengths above 16 Mb cover on average 4% of the BV and 11% of the LZ cow populations.

The distribution of ROH segments across the genome chromosomes was examined. ROH islands were detected on chromosomes 2 and 11 for BV and on chromosomes 10, 12, and 14 for LZ (Figure S3).

### 3.4. Genomic Inbreeding

**Figure 4:**
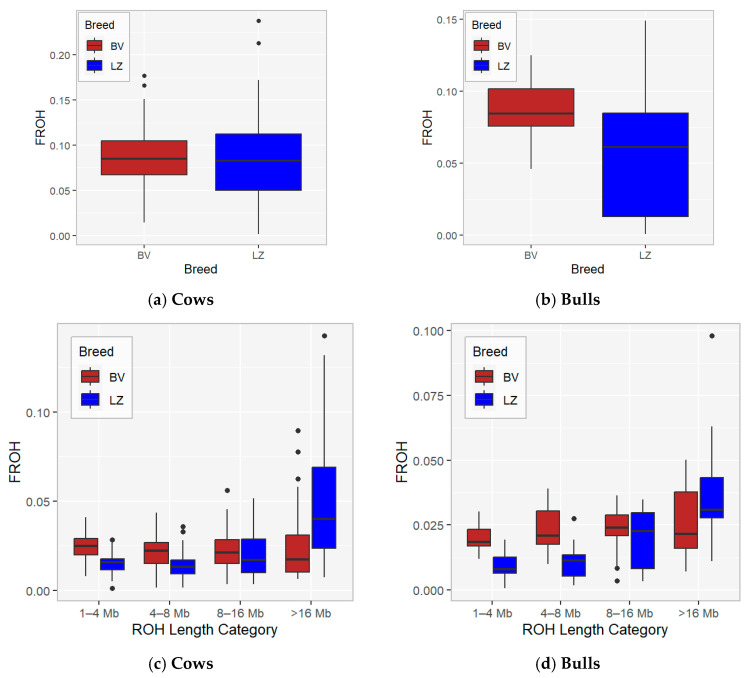
F_ROH_ by cow (**a**) and bull (**b**) data and F_ROH_ by ROH length category and cow (**c**) and bull (**d**) data.

Genomic inbreeding was analysed based on ROH length. Inbreeding was calculated using total ROH length (Figure 4a,b; Tables S3 and S4) and in four ROH length categories (Figure 4c,d; Tables S3 and S4). In our study, for inbreeding evaluation, total ROH length and ROH with minimum lengths of 1, 4, 8, and 16 Mb were used, which can be translated to 50, 12.5, 6.25, and 3.125 generations ago [24].

F_ROH_ statistics by ROH category within cows and bulls for the BV and LZ breeds are presented in Table 3 and Figure 4c,d. The average inbreeding coefficient for approximately three generations (F_ROH>16_) was 2.30% for BV and 4.87% for LZ cows. The current study demonstrates that inbreeding hardly changed over generations in the BV cow population and rapidly increased from 2.01% to 4.87% (F_ROH>16_ is higher compared with F_ROH<16_) in the LZ cow population (*p* < 0.05). The results showed that a few animals with high inbreeding coefficients in BV and LZ breeds have influenced the average value of recent inbreeding (F_ROH>16_).

In the bull population, recent inbreeding (F_ROH_) in the >16 Mb ROH category is 2.59% in BV and 3.85% in the LZ population; there is an increase in inbreeding from generation to generation in LZ population. The level of current inbreeding in LZ cows is higher compared with the bulls.

The relationships of genomic inbreeding between the bulls and their daughters (cows) were analysed. In the BV breed, the correlation was r_p_ = 0.198 (*n* = 42), whereas in the LZ breed, the correlation was higher r_p_ = 0.358 (*n* = 45, *p* < 0.05).

### 3.5. Cows’ Productivity and the Effect of Inbreeding on Cows’ Productivity

The first lactation milk productivity of the BV and LZ cows used in this study over a 305-day period is shown in Table 4 and Table S5. The analysis included cows that had finished their first lactation by the time of the SNP data analysis.

The average milk yield of the BV breed was 4013.1 kg, but in the LZ breed, the milk yield was 601 kg higher (*p* < 0.05). The milk yield of the most productive individual cows of the BV and LZ breeds exceeded 6000 kg. Both local cow breeds have a high milk fat and protein content: in the BV breed, it was, on average, 4.93% and 3.54%, while in the LZ breed, it was 4.57% and 3.39%. Due to the high fat and protein content, the average ECM (kg) of the cows of both breeds was higher than the average milk yield, indicating the suitability of the cows for the production of dairy products on farms.

In this study, we examined the relationship between genomic inbreeding and milk productivity; additionally, we analysed the effect of genomic inbreeding on the milk productivity of local cows in Latvia for the first time. The F_ROH_ for 78 BV breed cows ranged from 1.43% to 17.70%, with an average of 8.61%, and the F_ROH_ for 48 LZ breed cows was observed in a wider range, from 0.65% to 23.81%, with an average of 8.30% (Table 4).

**Figure 5:**
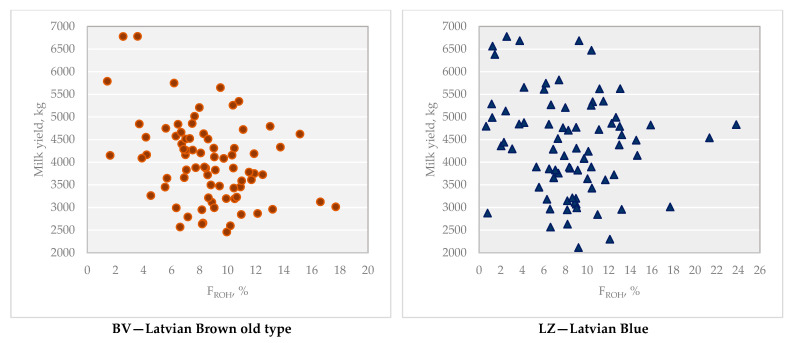
Milk yield depending on F_ROH_, % for BV and LZ breeds.

A negative phenotypic correlation was observed between F_ROH_ and milk yield for both breeds. In the BV breed, the correlation was weak but significant (r_p_ = −0.367; *p* < 0.01), whereas in the LZ breed, the correlation was not significant (r_p_ = −0.099) (Figure 5).

Inbreeding depression was observed in both BV and LZ cows. The linear regression coefficient showed a reduction in milk yield by 108.9 ± 31.56 kg (*p* < 0.01) with a 1% increase in F_ROH_ in the BV breed and 21.5 ± 31.54 kg in the LZ breed.

## 4. Discussion

Using high-density SNP genotype data, the genetic diversity of the Latvian Brown old type (BV) and Latvian Blue (LZ) local cattle populations was studied.

The results of our study demonstrate genetic distance and clear separation between the BV and LZ cattle breeds, and our results expand upon previously reported findings based on the PCA results [15]. Gudra et al. [15] indicated differences between breeds and that Latvian cow breeds are genetically distant from major European breeds; they also noted that Latvian Blue breed animals are clustered closer to European commercial breeds, particularly the Holstein breed. Population size and genetic management of the breeds can explain these differences. LB has been selected in Latvia for more than 100 years. Initially, Angler, Danish Red, and later other red breeds were used to develop and improve the LB breed. Since 2004, LB has been part of a conservation programme, and the decision was made to split the population into two parts—Latvian Brown new and old types—depending on the animals’ genetic background. Animals with 50% of LB genetic background and with Danish Red or Angler old-type ancestors are defined as genetic resources with the name Latvian Brown old type (the abbreviation BV has only been in use since 2024), and these animals are included in the analysis. The LZ breed cluster showed high distances between animals. Recovery of the LZ breed started around 30 years ago with a small number of surviving animals, and due to the small number of males in the population, Latvian Brown, Holstein, Lithuanian Light Grey, and Tyrol Grey breeds are the main breeds for crossbreeding in LZ [14].

Evaluation of observed heterozygosity (Ho) also was included in our analysis and there is slightly lower observed heterozygosity in the BV population in comparison with LZ. The reasons for the decrease in the level of heterozygosity or genetic variability may be the selection of animals according to economically important productivity traits or the isolation of the population. As shown in various other genomic studies, low-to-moderate heterozygosity is observed between local breeds [25,26,27]. In our study, both Latvian cattle breeds had moderate levels of heterozygosity, with values 0.397–0401 for BV and 0.403–0.413 for LZ. For a long time, the LB population was under selection pressure in comparison with LZ, and LB was selected for its milk productivity and conformation traits; as a result, there is slightly lower observed heterozygosity in the BV population (BV is part of the LB breed) in comparison with LZ. These results differ from those reported by Gudra et al. [15], where lower heterozygosity was observed. One possible explanation for the higher heterozygosity in our study could be the larger number of animals. Despite the differences in absolute values, both studies show that the breeds do not differ in heterozygosity and have comparable levels of genetic diversity.

Over the last decade, ROH began to be used in domestic animal studies [22,28]. The ROH profile of the BV and LZ breeds, characterized by 50 to 60% short ROH and 4–11% long ROH, indicates a mixture of ancient and recent inbreeding. Our results are in accordance with the investigation performed by Gudra et al. [15] that was carried out in Latvian local cow populations, where the majority of short ROHs were observed in the Latvian Blue and Latvian Brown populations [15]. The longest total ROH was detected in the LZ population (Figure 3), which could have been caused by mating between close relatives.

ROH islands were examined and were detected on chromosomes 2 and 11 for BV and on chromosomes 10, 12, and 14 for LZ. Although currently no selection is carried out for milk productivity in BV and LZ breeds, ROH islands, which have a confirmed impact on milk production traits, were identified. In many countries, ROH islands studies on commercial breeds were performed [29,30,31,32,33]. For example, Italian Holstein cows have an SNP on chromosome 2 related to milk yield, fat, and protein yield, while an SNP on chromosome 11 is related to milk yield and protein yield (LGB) [29]. Additionally, ROH islands on chromosome BTA11 were identified in a Chinese Holstein population and were associated with milk yield, fat yield, protein yield, and protein content [30]. The association between ROH on chromosome BTA14 and QTL influencing milk production traits has been widely reported, with particular emphasis on the DGAT1 gene locus, which has been shown to affect milk yield, as well as fat and protein yield and content, in Holstein cattle [29,32] and local Polish breeds [32].

Based on these ROH profiles, the inbreeding coefficient F_ROH_ was calculated. ROH inbreeding is more precise and does not suffer from limited pedigree depth [34], and it could be used to evaluate the level of inbreeding in local breeds or in populations with missing pedigree data or where pedigrees are not correct [24]. This approach also allows for the evaluation of ancient and recent inbreeding in the population; shorter ROHs (<4 Mb) indicate inbreeding from earlier generations (more than 12.5 generations ago), and longer ROHs (>16 Mb) indicate recent inbreeding from three generations ago [28]. The current study demonstrates that inbreeding hardly changed over generations in the BV cow and bull population and rapidly increased from 2.01% to 4.87% in the LZ cow and from 2.02% to 3.85% in the LZ bull population.

The estimates obtained for recent inbreeding in BV and LZ populations correspond to the inbreeding estimates of pedigrees found by Paura and Jonkus [14], where they were 2.61% and 5.20% for LB and LZ breeds, respectively. In the investigation performed by Gudra et al. [15], the inbreeding level based on the sum of all ROH taken together was higher in the Latvian Brown than in the Latvian Blue breed, and on F_ROH>50kb_, it was higher than 10% in both populations; the results in their study differ from ours, but this could be explained by the fact that they had a smaller number of animals.

Average F_ROH_ inbreeding values were reported by Mastrangelo et al. [25] in 32 cattle breeds, with moderate differences in F_ROH_ estimates between European breeds, where the authors observed several breeds with a small (<2%) and high (> 5%) inbreeding level in some local populations that have not undergone breeding programmes. According to the investigation performed by Senczuk et al. [35], average F_ROH_ inbreeding was lower among the local breeds (F_ROH_ = 1.4% for Original Brown from Italy and F_ROH_ = 1.7% for Cika) compared with highly selected breeds (F_ROH_ = 11.0% for Jersey and 10.5% for modern Brown). An investigation into Estonian populations [36] shows the higher F_ROH_ for Estonian Holstein (F_ROH_ = 11.5%) compared with Estonian Red (F_ROH_ = 4.4%), which is a local breed. The lowest F_ROH_ values were noted for native cattle breeds maintained in Poland [32]. The F_ROH>16_ in red cattle populations originating in Northern Europe ranges from 1.4% in Angler to 4.6% in Traditional Danish Red [37] due to a reduction in their population sizes and mating of related individuals. Studies on Tyrol Grey cattle have shown that local populations can contain animals with a high level of inbreeding [38].

In addition, recent F_ROH_ values differ between BV and LZ cows, and they are lower in the BV breed (*p* < 0.05). This can be explained, at least in part, by breeding organisations’ continued use of LB bull semen collected many years ago in the Latvian AI stations. The aim for local breeds is primarily conservation rather than genetic improvement. Many bulls used decades ago were genetically valuable, and old semen represents four unique genetic LB breed lineages. To reduce the level of inbreeding in the BV breed, for AI, breeders can not only use semen collected at the present period but also semen stored since the 1970s. Local breeds have a limited number of unrelated sires, and old semen leads to slow inbreeding accumulation and maintains genetic diversity.

The absence of systematic selection of the LZ breed in the previous century combined with the near-extinction of the breed has resulted in a higher recent inbreeding level compared with the BV population. A limited number of sires and potential historical bottlenecks influence the inbreeding level in LZ. Such factors form a higher genetic relationship among animals and accelerate the loss of genetic diversity.

Along with the diversity analysis of BV and LZ populations, cows’ productivity, as well as the effect of inbreeding on cows’ productivity was evaluated. Latvian local cow breeds are not characterised by high milk yields, but they have a high content of dry milk matter. An analysis performed in previous years of milk production in local breed cows shows that energy-corrected 305-day milk (ECM) ranged from 12.2 to 14.5 kg per productive life day for 20 years [39]. The milk yield of local cow breeds in other European countries is also generally lower than that of commercial breeds [40,41].

Pedigree-based and genome-based inbreeding levels and their impact on cow productivity have been extensively studied [42,43,44] in cow populations. We have performed a preliminary study about the effect of genomic inbreeding on the milk productivity in Latvia local cow population for the first time. Inbreeding depression was observed in both BV and LZ populations, where it was found that cow milk productivity decreases with an increase in the level of inbreeding. Similar conclusions about the decrease in milk yield with an increase in F_ROH_ by 1% have also been reached in studies performed in other countries. For example, in the German Holstein breed, milk yield decreased by 40.61 kg [45], and in Dutch Holstein–Friesian dairy cattle, the milk yield over 305 lactation days decreased by 36.3 kg [46]. In addition, in Italian Holstein cow populations, a 1% increase in inbreeding has been found to have a negative effect on milk yield (−61 kg) [47]. This study demonstrates that milk productivity in Latvian local populations is relatively low; their production level aligns with the productivity of local cattle breeds in other regions. This study also shows that genetic inbreeding has a negative influence on milk productivity in cows, while its influence on protein content is not significant. Because the number of animals in this study is small, it is reasonable to assume that these results should be considered preliminary; further studies with a larger dataset and data evaluation with mixed models are required.

Overall, our results demonstrate an overview of ROH patterns, inbreeding level, and productivity in BV and LZ populations. Continued monitoring of mating plans, the mating of non-related individuals, and control of inbreeding level will be essential to maintain genetic diversity in the Latvian local cow populations. Further studies on larger number of animals would strengthen our conclusions.

## 5. Conclusions

We conclude that the level of inbreeding calculated based on the ROH determines ancestral and recent inbreeding, and recent inbreeding from the ROH is in accordance with previously evaluated pedigree-based inbreeding estimates. It was found that cow milk productivity is related to genomic inbreeding, and milk productivity decreases with an increase in the level of inbreeding. Therefore, the control of inbreeding in populations is an important goal in breeding conservation programmes. We suggest that pedigree data and genotypes be collected continuously, and both be used in analyses of breed diversity. Nevertheless, moderate heterozygosity and inbreeding levels make preserving the genetic diversity of both breeds a priority.

## Figures and Tables

**Table 1:** Descriptive statistics of heterozygosity and minor allele frequency for BV and LZ breeds within cow and bull groups.

Breed	Cows		Bulls	
	Ho Mean (SD)	MAF Mean (SD)	Ho Mean (SD)	MAF Mean (SD)
BV	0.401 (0.014)	0.319 (0.122)	0.397 (0.009)	0.316 (0.129)
LZ	0.403 (0.023)	0.318 (0.124)	0.413 (0.019)	0.315 (0.131)

Note: BV—Latvian Brown; LZ—Latvian Blue; Ho—observed heterozygosity; MAF—minor allele frequency; SD—standard deviation.

**Table 2:** Descriptive statistics of individual ROH segments category for BV and LZ breeds within cow and bull data.

ROH Category ^1^	BV		LZ	
	ROH, kb Mean (SD)	ROH Count (Frequency)	ROH, kb Mean (SD)	ROH Count (Frequency)
Cows				
1–4 Mb	2158 (804)	2659 (0.63)	2073 (786)	1310 (0.60)
4–8 Mb	5637 (1123)	902 (0.22)	5820 (1134)	373 (0.17)
8–16 Mb	10,855 (2111)	465 (0.11)	11,734 (2186)	265 (0.12)
>16 Mb	24,790 (9632)	174 (0.04)	27,145 (11,037)	251 (0.11)
Bulls				
1–4 Mb	2410 (708)	414 (0.54)	2247 (757)	178 (0.50)
4–8 Mb	5777 (1123)	202 (0.26)	5667 (1160)	77 (0.22)
8–16 Mb	11,044 (2209)	104 (0.14)	10,967 (2065)	60 (0.17)
>16 Mb	26,193 (10,146)	42 (0.06)	27,115 (9174)	39 (0.11)

Note: BV—Latvian Brown; LZ—Latvian Blue; ROH—runs of homozygosity; ^1^ ROH 1–4 Mb; 4–8 Mb; 8–16 Mb, and >16 Mb translated to 50, 12.5, 6.25, and 3.125 generations ago.

**Table 3:** Descriptive statistics of genomic inbreeding coefficients by ROH (F_ROH_) category of the BV and LZ breeds.

ROH Category ^1^	BV		LZ	
	F_ROH_, % Mean (SD)	F_ROH_, % Range	F_ROH_,% Mean (SD)	F_ROH_,% Range
Cows				
1–4 Mb	2.44 (0.66)	0.80–4.09	1.51 (0.54)	0.12–2.83
4–8 Mb	2.16 (0.92)	0.18–4.34	1.36 (0.71)	0.17–3.59
8–16 Mb	2.18 (1.06)	0.36–5.60	2.01 ^a^ (1.19)	0.35–5.16
>16 Mb	2.30 * (1.70)	0.65–8.96	4.87 *^a^ (3.10)	0.73–14.29
Bulls				
1–4 Mb	2.00 (0.50)	1.20–3.01	0.89 (0.46)	0.06–1.93
4–8 Mb	2.33 (0.83)	0.99–3.91	1.09 (0.67)	0.17–2.75
8–16 Mb	2.30 (0.87)	0.35–3.64	2.02 (1.17)	0.34–3.49
>16 Mb	2.59 (1.46)	0.70–5.02	3.85 (2.42)	1.10–9.82

Note: BV—Latvian Brown; LZ—Latvian Blue; F_ROH_ the genomic inbreeding coefficients by ROH; between breeds *—*p* < 0.05; between generations a—*p* < 0.05; ^1^ ROH 1–4 Mb, 4–8 Mb, 8–16 Mb, and >16 Mb translated to 50, 12.5, 6.25, and 3.125 generations ago.

**Table 4:** Descriptive statistics of first lactation productivity traits of BV and LZ breeds and average F_ROH_, %.

Trait	BV (*n* = 78)		LZ (*n* = 48)	
	Mean (SD)	Range	Mean (SD)	Range
Milk yield, kg	4013.1 * (913.50)	2456–6780	4614.4 * (1104.92)	2115–6687
Fat content, %	4.93 (0.52)	3.78–6.44	4.57 (0.48)	3.67–5.66
Protein content, %	3.54 (0.26)	3.03–4.26	3.39 (0.23)	3.00–3.98
ECM, kg	4483.3 (938.31)	2876.5–7482.5	4941.8 (1303.35)	2377.6–7966.7
F_ROH_, %	8.61 (3.08)	1.43–17.70	8.30 (5.14)	0.65–23.81

Note: BV—Latvian Brown; LZ—Latvian Blue; 305-day milk yield; ECM—energy-corrected milk; *—*p* < 0.05.

## Data Availability

The data presented in this study are available on request from the corresponding author.

## Supplementary Materials

The following supporting information can be downloaded at: https://www.mdpi.com/article/10.3390/ani16010020/s1, Figure S1. Sampling locations for BV and LZ cows. Figure S2. PCA plot of PC1 vs. PC3 and PC2 vs. PC3 for the cows and bulls data set. Figure S3. Manhattan plot distribution of ROH islands on chromosomes: (a) in BV population and (b) in LZ population. Table S1. ROH segments length by ROH category in BV and LZ breeds’ bulls. Table S2. ROH segments length by ROH category in BV and LZ breeds’ cows. Table S3. F_ROH_ by total ROH and by ROH category in BV and LZ breeds’ bulls. Table S4. F_ROH_ by total ROH and by ROH category in BV and LZ breeds’ cows. Table S5. First lactation cows’ productivity and ROH-based inbreeding (F_ROH_) in BV and LZ breeds.

## References

[1] Regulation (EU) 2016/1012 of the European Parliament and of the Council of 8 June 2016 Clause 24 of Article 2. https://eur-lex.europa.eu/legal-content/EN/TXT/PDF/?uri=CELEX:32016R1012&qid=1689913000067.

[2] Breed Societies Maintaining Breeding Books for Purebred Breeding Animals. https://www.lad.gov.lv/lv/media/11452/download?attachment.

[3] Domestic Animal Diversity Information System (DAD-IS). https://www.fao.org/dad-is/browse-by-country-and-species/en/.

[4] European Regional Focal Point for Animal Genetic Resources (ERFP). https://www.animalgeneticresources.net/index.php/country/latvia/#1589182530764-d1c43160-c850.

[5] Latvian Brown Cow’s Conservation Program from 2019 and on (In Latvian). https://drive.google.com/file/d/1AMcfGytj9pJcqjllyEMNiIEDQrM8wX7-/view.

[6] Breeding Program of the Latvian Blue Cow Breed 2019–2029 (In Latvian). https://www.lad.gov.lv/lv/normativie-akti-par-dzivnieku-registraciju.

[7] FAO (2015). The Second Report on the State of the World’s Animal Genetic Resources for Food and Agriculture.

[8] Notter D.R. (1999). The importance of genetic diversity in livestock populations of the future. J. Anim. Sci..

[9] Reljanovic M., Ristov S., Curik C.V., Cacic M., Ferencakovic M., Curik I. (2015). Genealogical decomposition of the effective population size: A case study on croatian autochthonous cattle breeds. Poljoprivreda.

[10] Biscarini F., Mastrangelo S., Catillo G., Senczuk G., Ciampolini R. (2020). Insights into Genetic Diversity, Runs of Homozygosity and Heterozygosity-Rich Regions in Maremmana Semi-Feral Cattle Using Pedigree and Genomic Data. Animals.

[11] Fabbri M.C., Dadousis C., Bozzi R. (2020). Estimation of Linkage Disequilibrium and Effective Population Size in Three Italian Autochthonous Beef Breeds. Animals.

[12] Nyman S., Johansson A.M., Palucci V., Schönherz A.A., Guldbrandtsen B., Hinrichs D., de Koning D.J. (2022). Inbreeding and pedigree analysis of the European red dairy cattle. Genet. Sel. Evol..

[13] Marašinskienė Š., Šveistienė R., Razmaitė V., Račkauskaitė A., Juškienė V. (2023). Genetic Variability and Conservation Challenges in Lithuanian Dairy Cattle Populations. Animals.

[14] Paura L., Jonkus D. (2020). Inbreeding Evaluation in Latvian Local Cattle Breeds. Acta Fytotech. Zootech..

[15] Gudra D., Valdovska A., Jonkus D., Galina D., Kairisa D., Ustinova M., Viksne K., Fridmanis D., Kalnina I. (2023). Genomic Characterization and Initial Insight into Mastitis-Associated SNP Profiles of Local Latvian Bos taurus Breeds. Animals.

[16] Chang C.C., Chow C.C., Tellier L.C., Vattikuti S., Purcell S.M., Lee J.J. (2015). Second-Generation PLINK: Rising to the Challenge of Larger and Richer Datasets. GigaScience.

[17] PLINK: Whole Genome Data Analysis Toolset. http://zzz.bwh.harvard.edu/plink/.

[18] Meyermans R., Gorssen W., Buys N., Janssens S. (2020). How to Study Runs of Homozygosity Using PLINK? A Guide for Analyzing Medium Density SNP Data in Livestock and Pet Species. BMC Genom..

[19] R Core Team (2025). R: A Language and Environment for Statistical Computing.

[20] Purfield D.C., Berry D.P., McParland S., Bradley D.G. (2012). Runs of Homozygosity and Population History in Cattle. BMC Genet..

[21] McQuillan R., Leutenegger A.-L., Abdel-Rahman R., Franklin C.S., Pericic M., Barac-Lauc L., Smolej-Narancic N., Janicijevic B., Polasek O., Tenesa A. (2008). Runs of Homozygosity in European Populations. Am. J. Hum. Genet..

[22] Ferenčaković M., Hamzic E., Gredler B., Curik I., Solkner J. (2011). Runs of homozygosity reveal genome-wideautozygosity in the Austrian fleckvieh cattle. Agric. Conspec. Sci..

[23] Marras G., Gaspa G., Sorbolini S., Dimauro C., Ajmone-Marsan P., Valentini A., Williams J.L., Macciotta N.P.P. (2014). Analysis of Runs of Homozygosity and Their Relationship with Inbreeding in Five Cattle Breeds Farmed in Italy. Anim. Genet..

[24] Curik I., Ferenčaković M., Sölkner J. (2014). Inbreeding and Runs of Homozygosity: A Possible Solution to an Old Problem. Livest. Sci..

[25] Mastrangelo S., Ciani E., Marsan P.A., Bagnato A., Battaglini L., Bozzi R., Carta A., Catillo G., Cassandro M., Casu S. (2018). Conservation status and historical relatedness of Italian cattle breeds. Genet. Sel. Evol..

[26] Kasarda R., Vostrý L., Vostrá-Vydrová H., Candráková K., Moravčíková N. (2021). Food Resources Biodiversity: The Case of Local Cattle in Slovakia. Sustainability.

[27] Bayraktar M., Cebeci Z., Gökçe G. (2024). Analysing the Genetic Diversity and Population Structure of Five Native Turkish Cattle Breeds Using SNP Data. Reprod. Domest. Anim..

[28] Ferenčaković M., Hamzić E., Gredler B., Solberg T.R., Klemetsdal G., Curik I., Sölkner J. (2012). Estimates of Autozygosity Derived from Runs of Homozygosity: Empirical Evidence from Selected Cattle Populations. J. Anim. Breed. Genet..

[29] Fontanesi L., Calò D.G., Galimberti G., Negrini R., Marino R., Nardone A., Ajmone-Marsan P., Russo V. (2014). A candidate gene association study for nine economically important traits in Italian Holstein cattle. Anim. Genet..

[30] Jiang J., Liu L., Gao Y., Shi L., Li Y., Liang W., Sun D. (2019). Determination of genetic associations between indels in 11 candidate genes and milk composition traits in Chinese Holstein population. BMC Genet..

[31] Ristanic M., Zorc M., Glavinic U., Stevanovic J., Blagojevic J., Maletic M., Stanimirovic Z. (2024). Genome-Wide Analysis of Milk Production Traits and Selection Signatures in Serbian Holstein-Friesian Cattle. Animals.

[32] Szmatoła T., Gurgul A., Jasielczuk I., Ząbek T., Ropka-Molik K., Litwińczuk Z., Bugno-Poniewierska M. (2019). A Comprehensive Analysis of Runs of Homozygosity of Eleven Cattle Breeds Representing Different Production Types. Animals.

[33] Wirth A., Duda J., Emmerling R., Götz K.U., Birkenmaier F., Distl O. (2024). Analyzing Runs of Homozygosity Reveals Patterns of Selection in German Brown Cattle. Genes.

[34] Zhang Q., Calus M.P., Guldbrandtsen B., Lund M.S., Sahana G. (2015). Estimation of Inbreeding Using Pedigree, 50k SNP Chip Genotypes and Full Sequence Data in Three Cattle Breeds. BMC Genet..

[35] Senczuk G., Mastrangelo S., Ciani E., Battaglini L., Cendron F., Ciampolini R., Crepaldi P., Mantovani R., Bongioni G., Pagnacco G. (2020). The Genetic Heritage of Alpine Local Cattle Breeds Using Genomic SNP Data. Genet. Sel. Evol..

[36] Värv S., Põlluäär T., Sild E., Viinalass H., Kaart T. (2024). Genetic Variation and Composition of Two Commercial Estonian Dairy Cattle Breeds Assessed by SNP Data. Animals.

[37] Schmidtmann C., Schönherz A.A., Guldbrandtsen B., Marjanovic J., Calus M.P.L., Hinrichs D., Thaller G. (2021). Assessing the Genetic Background and Genomic Relatedness of Red Cattle Populations Originating from Northern Europe. Genet. Sel. Evol..

[38] Mészáros G., Boison S.A., Pérez O’Brien A.M., Ferenčaković M., Curik I., Da Silva M.V., Utsunomiya Y.T., Garcia J.F., Sölkner J. (2015). Genomic Analysis for Managing Small and Endangered Populations: A Case Study in Tyrol Grey Cattle. Front. Genet..

[39] Jonkus D., Paura L., Cielava L. (2020). Longevity and Milk Production Efficiency of Latvian Local Breeds during Last Decades. Agron. Res..

[40] Gandini G., Maltecca C., Pizzi F., Bagnato A., Rizzi R. (2007). Comparing Local and Commercial Breeds on Functional Traits and Profitability: The Case of Reggiana Dairy Cattle. J. Dairy Sci..

[41] Bieber A., Wallenbeck A., Leiber F., Fuerst-Waltl B., Winckler C., Gullstrand P., Walczak J., Wójcik P., Neff A.S. (2019). Production Level, Fertility, Health Traits, and Longevity in Local and Commercial Dairy Breeds under Organic Production Conditions in Austria, Switzerland, Poland, and Sweden. J. Dairy Sci..

[42] Bjelland D.W., Weigel K.A., Vukasinovic N., Nkrumah J.D. (2013). Evaluation of Inbreeding Depression in Holstein Cattle Using Whole-Genome SNP Markers and Alternative Measures of Genomic Inbreeding. J. Dairy Sci..

[43] Dezetter C., Leclerc H., Mattalia S., Barbat A., Boichard D., Ducrocq V. (2015). Inbreeding and Crossbreeding Parameters for Production and Fertility Traits in Holstein, Montbéliarde, and Normande Cows. J. Dairy Sci..

[44] Bezdicek J., Nesvadbová A., Louda F. (2020). Relationship between inbreeding and daily milk production in Holstein cows. Slovak J. Anim. Sci..

[45] Mugambe J., Ahmed R., Thaller G., Schmidtmann C. (2024). Impact of Inbreeding on Production, Fertility, and Health Traits in German Holstein Dairy Cattle Utilizing Various Inbreeding Estimators. J. Dairy Sci..

[46] Doekes H.P., Veerkamp R.F., Bijma P., de Jong G., Hiemstra S.J., Windig J.J. (2019). Inbreeding Depression due to Recent and Ancient Inbreeding in Dutch Holstein–Friesian Dairy Cattle. Genet. Sel. Evol..

[47] Ablondi M., Summer A., Stocco G., Finocchiaro R., van Kaam J.T., Cassandro M., Dadousis C., Sabbioni A., Cipolat-Gotet C. (2023). The Role of Inbreeding Depression on Productive Performance in the Italian Holstein Breed. J. Anim. Sci..

