# Supplementary material for: Genomic Analysis of Latvian Brown Old Type and Latvian Blue Local Dairy Cattle Breeds Using SNP Data
Source: Animals (Basel). 2025 Dec 20;16(1):20. doi: 10.3390/ani16010020 (PMC12784749; doi:10.3390/ani16010020)
Supplement: Supplementary file 1 [file animals-16-00020-s001.zip › Figure_S3.pdf]

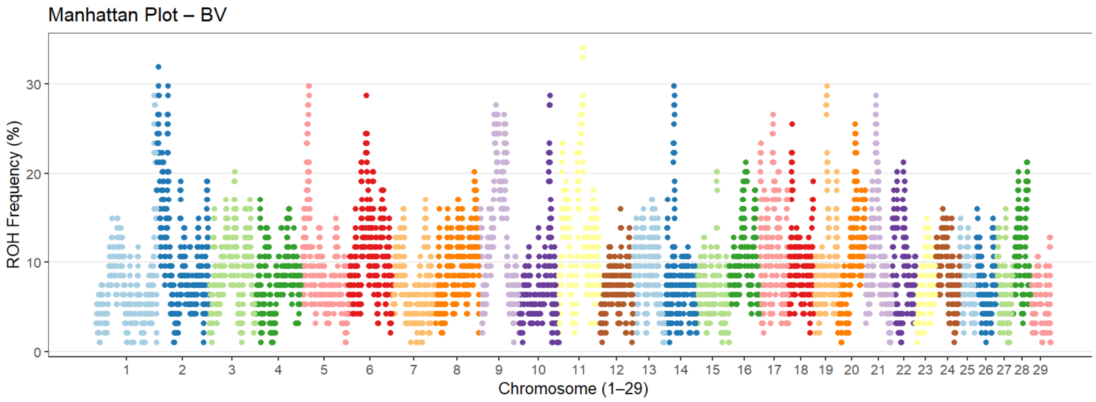

a) BV population

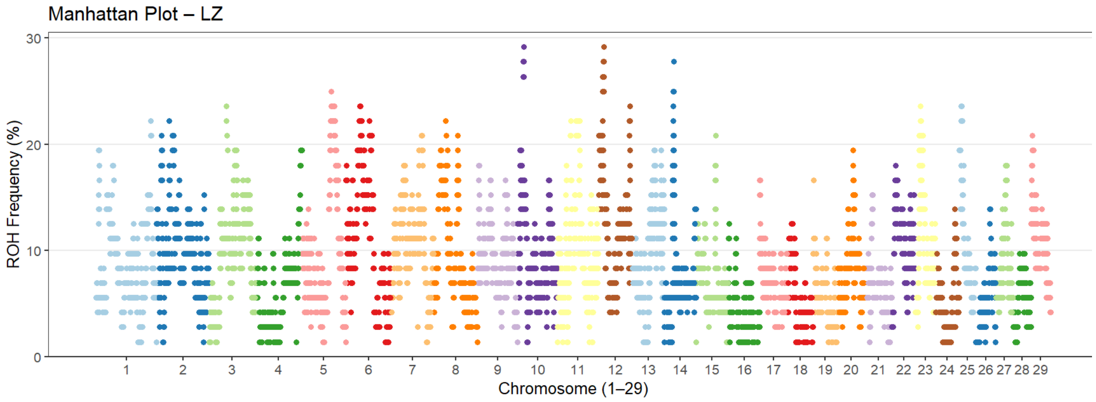

b) LZ population

Figure S3. Manhattan plot distribution of ROH islands on chromosomes: a) in BV population and b) in LZ population.
