# Supplementary material for: Genomic Analysis of Latvian Brown Old Type and Latvian Blue Local Dairy Cattle Breeds Using SNP Data
Source: Animals (Basel). 2025 Dec 20;16(1):20. doi: 10.3390/ani16010020 (PMC12784749; doi:10.3390/ani16010020)
Supplement: Supplementary file 1 [file animals-16-00020-s001.zip › Table S1.pdf]

Table S1. ROH segments length by ROH category in BV and LZ breeds' bulls.

Bull code	Breed	ROH 1–4 Mb		ROH 4–8 Mb		ROH 8–16 Mb		ROH > 16 Mb	
		Frequency	Total ROH, KB	Frequency	Total ROH, KB	Frequency	Total ROH, KB	Frequency	Total ROH, KB
1	BV	18	40,306	7	43,739	1	8,739	4	94,459
2	BV	27	69,850	13	75,587	2	20,788	2	50,406
3	BV	29	69,718	11	69,203	7	75,341	1	35,582
4	BV	12	30,040	17	97,840	5	52,425		
5	BV	16	40,097	10	55,829	4	50,436	3	120,005
6	BV	18	44,381	7	43,562	9	91,036	4	122,177
7	BV	26	62,776	15	76,768	5	56,469	2	54,925
8	BV	19	46,070	4	24,768	6	65,368	3	50,449
9	BV	20	42,966	7	39,137	3	32,687		
10	BV	16	43,097	9	47,719	5	57,927	2	57,170
11	BV	24	57,864	10	53,542	5	53,878	1	24,185
12	BV	30	75,180	7	45,244	7	72,193	5	119,886
13	BV	21	47,782	8	51,110	2	22,705	2	56,118
14	BV	21	48,764	15	81,645	6	66,289		
15	BV	14	31,735	6	34,823	7	84,354	2	39,872
16	BV	26	59,146	7	44,489	7	78,145	1	17,601
17	BV	18	46,439	11	66,554	6	70,483	2	35,221
18	BV	25	56,747	16	95,946	7	72,022	2	53,987
19	BV	19	44,594	14	78,671	5	55,099	2	42,689
20	BV	15	40,107	8	40,702	5	62,235	4	125,386
	Total	414	997,659	202	1,166,876	104	1,148,620	42	1,100,117
	Mean*		2,410		5,777		11,044		26,193
	SD*		708		1,123		2,219		10,146
	Min*		1,048		4,019		8,061		16,001
	Max*		3,983		7,984		15,918		65,546

Bull code	Breed	ROH 1–4 Mb		ROH 4–8 Mb		ROH 8–16 Mb		ROH > 16 Mb	
		Frequency	Total ROH, KB	Frequency	Total ROH, KB	Frequency	Total ROH, KB	Frequency	Total ROH, KB
21	LZ	8	19,558	2	10,665	1	8,452		
22	LZ	10	22,661	6	30,869	2	20,370	1	27,512
23	LZ	4	9,174	7	36,306	6	65,104	3	102,807
24	LZ	12	28,142	4	22,907	6	74,039	1	40,409
25	LZ	7	15,419						
26	LZ	8	17,449	4	25,522	7	84,576	8	245,376
27	LZ	8	17,485	2	12,694				
28	LZ	16	30,400	12	68,858	9	87,294	3	67,414
29	LZ	7	12,547	1	4,266				
30	LZ	14	31,937	7	42,985	5	57,616	3	77,203
31	LZ	14	34,136	6	30,495	4	40,160	6	157,528
32	LZ	13	32,138	2	10,802	2	19,077	3	78,361
33	LZ	8	19,996	5	32,703	8	87,147	5	113,186
34	LZ	21	48,159	5	30,452	4	48,581	3	76,943
35	LZ	7	16,765	8	48,245	5	56,550	3	70,737
36	LZ	15	33,788	3	15,217	1	9,075		
37	LZ	1	1,397						
38	LZ	5	8,853	3	13,361				
	Total	178	400,006	77	436,346	60	658,039	39	1,057,474
	Mean*		2,247		5,667		10,967		27,115
	SD*		757		1,160		2,065		9,174
	Min*		1,019		4,002		8,092		16,541
	Max*		3,977		7,987		15,973		51,637

Note: * Calculations were conducted based on the full dataset.
