# Supplementary material for: Genomic Analysis of Latvian Brown Old Type and Latvian Blue Local Dairy Cattle Breeds Using SNP Data
Source: Animals (Basel). 2025 Dec 20;16(1):20. doi: 10.3390/ani16010020 (PMC12784749; doi:10.3390/ani16010020)
Supplement: Supplementary file 1 [file animals-16-00020-s001.zip › Table S2.pdf]

Table S2. ROH segments length by ROH category in BV and LZ breeds' cows.

Cow code	Breed	ROH 1–4 Mb		ROH 4–8 Mb		ROH 8–16 Mb		ROH > 16 Mb	
		Frequency	Total ROH, KB	Frequency	Total ROH, KB	Frequency	Total ROH, KB	Frequency	Total ROH, KB
1	BV	12	22,203	1	4,423	1	9,001		
2	BV	25	50,489	8	48,394	3	26,837	6	136,247
3	BV	37	83,466	12	62,912	6	70,779	1	19,994
4	BV	30	66,516	7	34,913	6	60,164		
5	BV	16	32,126	6	31,365	3	29,136		
6	BV	25	58,986	7	35,720	7	75,700	1	29,128
7	BV	22	40,955	8	41,208	5	56,261	1	16,163
8	BV	24	49,977	1	4,412	1	9,533		
9	BV	24	50,630	11	67,688	7	63,988	1	22,346
10	BV	30	61,822	13	80,047	3	40,544		
11	BV	34	74,671	18	108,587	7	72,885	2	40,834
12	BV	29	61,073	13	71,936	7	87,768	7	194,408
13	BV	34	76,189	6	30,219	6	68,462		
14	BV	32	60,668	18	97,102	5	53,292	3	77,068
15	BV	30	69,513	9	48,936	6	70,892	1	17,795
16	BV	29	68,183	7	41,068	4	42,430	2	35,162
17	BV	23	47,757	11	61,816	6	58,569		
18	BV	36	86,774	14	75,868	5	53,802	2	46,160
19	BV	27	57,523	11	65,416	5	53,200	1	17,003
20	BV	31	74,790	6	32,536			1	34,624
21	BV	25	54,592	7	38,848	7	76,040	2	90,600
22	BV	32	75,559	10	57,889	3	41,647		
23	BV	28	57,301	9	47,973	3	28,251	1	24,951
24	BV	29	59,782	18	108,542	2	17,318	2	49,813
25	BV	40	85,535	13	77,267	8	92,596	2	47,862

Cow code	Breed	ROH 1–4 Mb		ROH 4–8 Mb		ROH 8–16 Mb		ROH > 16 Mb	
		Frequency	Total ROH, KB	Frequency	Total ROH, KB	Frequency	Total ROH, KB	Frequency	Total ROH, KB
26	BV	25	48,654	8	40,534	5	52,109	2	78,851
27	BV	32	70,875	12	66,592	3	29,599		
28	BV	24	54,752	7	37,851	11	111,421	1	22,346
29	BV	26	47,460	5	29,518	2	20,420		
30	BV	36	69,175	12	68,667	8	82,352	2	45,845
31	BV	30	63,980	11	67,463	7	67,314	4	145,103
32	BV	25	61,510	14	79,238	1	10,113	1	28,231
33	BV	9	20,079	4	23,056	2	18,099	2	43,304
34	BV	18	42,968	2	14,016	2	20,302	2	35,906
35	BV	31	69,532	12	65,117	7	88,081	5	155,955
36	BV	24	50,059	10	61,538	4	47,209	1	16,367
37	BV	24	56,379	9	53,172	9	92,563		
38	BV	33	75,453	11	52,733	5	48,307	4	93,479
39	BV	27	55,156	11	69,318	7	72,263	1	17,576
40	BV	36	75,081	7	40,826	5	60,783	2	38,265
41	BV	28	62,508	12	73,424	5	52,417	4	86,684
42	BV	36	73,153	11	60,725	10	113,572		
43	BV	29	61,993	8	36,592	5	63,658	2	43,760
44	BV	43	102,317	6	40,031	8	88,982	1	17,134
45	BV	17	37,584	11	58,520	2	18,874	2	50,318
46	BV	38	85,826	12	68,798	5	59,275	2	60,336
47	BV	41	89,497	14	77,052	6	68,324	4	95,203
48	BV	35	78,575	12	64,975	8	96,986		
49	BV	38	79,723	12	60,471	5	48,986	4	103,062
50	BV	20	39,569	7	39,815	4	41,631	1	17,529
51	BV	42	82,907	5	29,746	5	57,167	2	41,073
52	BV	30	63,843	8	40,324	5	52,277	1	16,322

Cow code	Breed	ROH 1–4 Mb		ROH 4–8 Mb		ROH 8–16 Mb		ROH > 16 Mb	
		Frequency	Total ROH, KB	Frequency	Total ROH, KB	Frequency	Total ROH, KB	Frequency	Total ROH, KB
53	BV	27	55,746	10	56,445	2	25,981	1	38,248
54	BV	24	50,864	6	36,237	6	73,848	2	60,205
55	BV	34	71,451	11	63,652	4	46,567	1	40,262
56	BV	36	88,088	7	41,648	6	62,030	1	24,004
57	BV	32	67,230	12	63,778	4	40,096	2	37,624
58	BV	29	64,026	16	89,555	6	64,908	7	223,956
59	BV	25	53,264	15	93,161	6	76,682	2	37,907
60	BV	30	63,469	11	59,512	1	9,445	5	93,673
61	BV	25	48,038	10	55,786	4	46,298	3	78,000
62	BV	37	86,803	15	87,535	8	87,862	2	50,257
63	BV	21	39,380	17	81,776	2	26,443	1	19,873
64	BV	26	58,915	8	43,636	5	50,678	1	18,446
65	BV	32	63,642	6	31,370	6	65,814	2	43,264
66	BV	29	61,914	7	34,331	4	51,956	4	129,316
67	BV	41	93,579	18	100,347	8	83,910	2	48,322
68	BV	26	48,942	3	15,659	4	42,305		
69	BV	16	33,916	6	30,094	5	57,180	1	26,749
70	BV	28	60,838	9	53,894	3	29,982		
71	BV	17	39,116	16	98,099	8	94,254	4	76,711
72	BV	34	71,312	8	47,184	5	54,621		
73	BV	17	37,849	3	17,862	4	40,963	3	60,113
74	BV	37	84,486	11	61,130	5	46,595	5	138,527
75	BV	37	77,201	11	59,547	4	42,359	3	111,537
76	BV	32	75,054	8	44,552	3	30,472	1	31,374
77	BV	20	44,285	4	21,880	3	25,901	1	17,598
78	BV	29	64,001	5	34,210	8	80,710	2	40,935
79	BV	34	73,354	9	49,748	8	73,984	2	38,380

Cow code	Breed	ROH 1–4 Mb		ROH 4–8 Mb		ROH 8–16 Mb		ROH > 16 Mb	
		Frequency	Total ROH, KB	Frequency	Total ROH, KB	Frequency	Total ROH, KB	Frequency	Total ROH, KB
80	BV	12	23,136	5	28,068	1	12,731	1	25,657
81	BV	17	42,465	5	25,143	3	32,926	2	39,423
82	BV	23	40,368	10	55,462	1	9,067	4	82,734
83	BV	35	76,712	12	56,845	5	51,004	2	73,228
84	BV	26	57,589	11	65,236	7	88,881	3	85,749
85	BV	33	62,365	8	43,880	5	51,688		
86	BV	29	58,112	10	56,753	6	67,337	3	72,580
87	BV	19	40,214	9	48,136	2	17,344		
88	BV	16	33,968	1	6,817				
89	BV	30	63,461	10	60,567	5	50,657	1	16,374
90	BV	32	71,433	9	54,443	8	85,685	3	61,372
91	BV	27	63,921	12	67,087	1	13,569	4	80,711
92	BV	30	65,142	10	65,026	13	140,111	3	55,409
93	BV	26	57,599	16	99,942	6	66,988	1	18,517
94	BV	28	59,426	15	83,456	5	53,027	3	63,552
	Total	2,659	5,738,358	902	5,084,682	462	5,015,044	174	4,313,393
	Mean*		2,158		5,637		10,855		24,790
	SD*		804		1,123		2,111		9,632
	Min*		1,004		4,012		8,000		16,042
	Max*		3,998		7,995		15,950		77,220

Note: * Calculations were conducted based on the full dataset.

Cow code	Breed	ROH 1–4 Mb		ROH 4–8 Mb		ROH 8–16 Mb		ROH > 16 Mb	
		Frequency	Total ROH, KB	Frequency	Total ROH, KB	Frequency	Total ROH, KB	Frequency	Total ROH, KB
95	LZ	15	35,185	10	59,437	6	72,083	4	95,128
96	LZ	23	50,986	6	30,971	8	82,312	2	52,626
97	LZ	10	18,118	1	7,569				
98	LZ	9	19,182	1	6,499	2	19,533	3	58,603
99	LZ	19	34,824	4	23,913	2	26,687	4	121,180
100	LZ	22	40,672	3	15,856	1	10,547	6	157,671
101	LZ	24	47,697	11	69,894	8	100,317	6	177,026
102	LZ	22	48,119	7	36,719	6	66,303	6	165,576
103	LZ	22	44,018	6	35,823	7	89,310	3	62,232
104	LZ	28	56,619	8	52,613	10	128,941	13	357,131
105	LZ	10	18,484	1	4,271				
106	LZ	20	42,885	3	21,650	3	34,593	8	227,512
107	LZ	30	59,767	6	35,256	7	84,416	5	99,329
108	LZ	16	30,663	6	32,846	3	31,318	4	99,364
109	LZ	11	19,604						
110	LZ	9	16,174						
111	LZ	16	34,292	5	27,245	1	14,995		
112	LZ	33	70,767	7	38,632	6	64,791	3	77,874
113	LZ	19	42,970	4	23,509	6	73,936	7	184,027
114	LZ	20	42,856	5	28,138	8	96,256	4	93,028
115	LZ	19	41,552	9	55,424	7	71,789	6	228,351
116	LZ	22	47,629	9	51,539	9	102,241	7	228,881
117	LZ	15	30,423	6	34,567	6	72,095	2	57,291
118	LZ	24	50,958	5	25,879	1	8,798	4	103,006
119	LZ	26	61,087	8	48,736	5	47,277	5	132,524
120	LZ	14	26,534	4	22,802	5	51,296	1	31,832
121	LZ	25	55,989	5	34,041	4	55,001	2	59,278

Cow code	Breed	ROH 1–4 Mb		ROH 4–8 Mb		ROH 8–16 Mb		ROH > 16 Mb	
		Frequency	Total ROH, KB	Frequency	Total ROH, KB	Frequency	Total ROH, KB	Frequency	Total ROH, KB
122	LZ	29	66,928	13	82,287	7	74,826		
123	LZ	16	38,652	9	47,946	1	10,758	3	64,846
124	LZ	22	45,137	7	46,686	7	91,404	3	76,483
125	LZ	23	45,467	8	42,640	4	43,406	1	25,658
126	LZ	21	43,290	7	40,369	3	34,890	7	175,317
127	LZ	20	36,611	6	33,838	3	35,123	4	91,585
128	LZ	20	40,754	7	40,970	2	21,733	4	99,793
129	LZ	27	58,455	5	24,012	9	106,500	7	177,230
130	LZ	16	35,482	6	32,040	6	67,937	4	94,574
131	LZ	21	44,017	5	27,608	3	30,458	7	200,611
132	LZ	17	35,428	4	19,478	4	49,098	1	33,574
133	LZ	17	39,821	5	27,197	7	83,295		
134	LZ	18	37,141	5	33,665	4	48,429	8	210,946
135	LZ	22	51,430	7	43,355	8	102,866	2	53,199
136	LZ	30	64,282	9	58,670	5	61,865	5	148,960
137	LZ	19	40,908	3	17,340	3	38,559	4	124,698
138	LZ	10	21,090	1	7,854				
139	LZ	12	21,520	2	12,638	2	23,198		
140	LZ	16	31,862	3	13,314	1	15,621	1	43,083
141	LZ	12	24,411			1	10,884	1	26,095
142	LZ	13	25,429						
143	LZ	20	41,725	4	24,328	5	64,109	5	157,613
144	LZ	21	44,386	8	46,920	3	40,668	6	131,055
145	LZ	25	47,907	15	89,647	6	66,484	12	329,179
146	LZ	15	29,632	1	7,052	1	14,131		
147	LZ	12	25,789	3	15,968	2	21,505	7	300,388
148	LZ	17	38,477	11	60,297	2	22,693	2	40,193

Cow code	Breed	ROH 1–4 Mb		ROH 4–8 Mb		ROH 8–16 Mb		ROH > 16 Mb	
		Frequency	Total ROH, KB	Frequency	Total ROH, KB	Frequency	Total ROH, KB	Frequency	Total ROH, KB
149	LZ	10	14,894						
150	LZ	20	37,194	7	35,479	3	36,557	2	57,118
151	LZ	12	22,980	1	4,226	1	9,105		
152	LZ	22	45,857	5	24,769	6	74,650	2	39,588
153	LZ	18	35,166	4	23,214	1	14,396	5	179,986
154	LZ	13	26,275	5	25,986	3	37,383	7	217,325
155	LZ	18	41,658	6	31,104	3	37,466	5	97,979
156	LZ	20	43,758	7	41,074	4	41,525	1	37,939
157	LZ	7	12,875					1	18,292
158	LZ	16	35,007	8	47,571	4	40,120	8	171,782
159	LZ	21	43,376	7	41,170	7	73,162	4	75,512
160	LZ	13	21,318	3	19,746	5	57,516	5	131,640
161	LZ	16	30,254						
162	LZ	2	2,993						
163	LZ	23	41,857	7	39,150	2	28,003	3	74,333
164	LZ	15	31,480	7	40,308	2	22,196		
165	LZ	21	43,890	4	25,377	2	27,589	4	109,593
166	LZ	9	20,568	8	51,813	2	24,480	5	127,693
	Total	1,310	2,715,487	373	2,170,934	265	3,109,423	251	6,813,332
	Mean*		2,073		5,820		11,734		27,145
	SD*		786		1,134		2,186		11,037
	Min*		1,001		4,014		8,026		16,112
	Max*		3,989		7,995		15,964		102,385

Note: * Calculations were conducted based on the full dataset.
