# Supplementary material for: Genomic Analysis of Latvian Brown Old Type and Latvian Blue Local Dairy Cattle Breeds Using SNP Data
Source: Animals (Basel). 2025 Dec 20;16(1):20. doi: 10.3390/ani16010020 (PMC12784749; doi:10.3390/ani16010020)
Supplement: Supplementary file 1 [file animals-16-00020-s001.zip › Table S3.pdf]

Table S3. F_{ROH} by total ROH and by ROH category for BV and LZ breeds' bulls.

Bull code	Breed	ROH 1–4 Mb		ROH 4–8 Mb		ROH 8–16 Mb		ROH > 16 Mb		Total	
		Total ROH, KB	F_{ROH}	Total ROH, KB	F_{ROH}	Total ROH, KB	F_{ROH}	Total ROH, KB	F_{ROH}	Total ROH, KB	F_{ROH}
1	BV	40,306	0.0161	43,739	0.0175	8,739	0.0035	94,459	0.0378	187,242	0.0749
2	BV	69,850	0.0279	75,587	0.0302	20,788	0.0083	50,406	0.0202	216,631	0.0867
3	BV	69,718	0.0279	69,203	0.0277	75,341	0.0301	35,582	0.0142	249,844	0.0999
4	BV	30,040	0.0120	97,840	0.0391	52,425	0.0210			180,305	0.0721
5	BV	40,097	0.0160	55,829	0.0223	50,436	0.0202	120,005	0.0480	266,367	0.1065
6	BV	44,381	0.0178	43,562	0.0174	91,036	0.0364	122,177	0.0489	301,156	0.1205
7	BV	62,776	0.0251	76,768	0.0307	56,469	0.0226	54,925	0.0220	250,937	0.1004
8	BV	46,070	0.0184	24,768	0.0099	65,368	0.0261	50,449	0.0202	186,654	0.0747
9	BV	42,966	0.0172	39,137	0.0157	32,687	0.0131			114,790	0.0459
10	BV	43,097	0.0172	47,719	0.0191	57,927	0.0232	57,170	0.0229	205,913	0.0824
11	BV	57,864	0.0231	53,542	0.0214	53,878	0.0216	24,185	0.0097	189,469	0.0758
12	BV	75,180	0.0301	45,244	0.0181	72,193	0.0289	119,886	0.0480	312,502	0.1250
13	BV	47,782	0.0191	51,110	0.0204	22,705	0.0091	56,118	0.0224	177,715	0.0711
14	BV	48,764	0.0195	81,645	0.0327	66,289	0.0265			196,699	0.0787
15	BV	31,735	0.0127	34,823	0.0139	84,354	0.0337	39,872	0.0159	190,785	0.0763
16	BV	59,146	0.0237	44,489	0.0178	78,145	0.0313	17,601	0.0070	199,381	0.0798
17	BV	46,439	0.0186	66,554	0.0266	70,483	0.0282	35,221	0.0141	218,697	0.0875
18	BV	56,747	0.0227	95,946	0.0384	72,022	0.0288	53,987	0.0216	278,702	0.1115
19	BV	44,594	0.0178	78,671	0.0315	55,099	0.0220	42,689	0.0171	221,053	0.0884
20	BV	40,107	0.0160	40,702	0.0163	62,235	0.0249	125,386	0.0502	268,429	0.1074
		Min	0.0120		0.0099		0.0035		0.0070		0.0459
		Max	0.0301		0.0391		0.0364		0.0502		0.1250
		Mean	0.0200		0.0233		0.0230		0.0259		0.0883
		SD	0.0050		0.0083		0.0087		0.0146		0.0194

Bull code	Breed	ROH 1–4 Mb		ROH 4–8 Mb		ROH 8–16 Mb		ROH > 16 Mb		Total	
		Total ROH, KB	F_{ROH}	Total ROH, KB	F_{ROH}	Total ROH, KB	F_{ROH}	Total ROH, KB	F_{ROH}	Total ROH, KB	F_{ROH}
21	LZ	19,558	0.0078	10,665	0.0043	8,452	0.0034			38,676	0.0155
22	LZ	22,661	0.0091	30,869	0.0123	20,370	0.0081	27,512	0.0110	101,413	0.0406
23	LZ	9,174	0.0037	36,306	0.0145	65,104	0.0260	102,807	0.0411	213,390	0.0854
24	LZ	28,142	0.0113	22,907	0.0092	74,039	0.0296	40,409	0.0162	165,497	0.0662
25	LZ	15,419	0.0062							15,419	0.0062
26	LZ	17,449	0.0070	25,522	0.0102	84,576	0.0338	245,376	0.0982	372,923	0.1492
27	LZ	17,485	0.0070	12,694	0.0051					30,179	0.0121
28	LZ	30,400	0.0122	68,858	0.0275	87,294	0.0349	67,414	0.0270	253,965	0.1016
29	LZ	12,547	0.0050	4,266	0.0017					16,813	0.0067
30	LZ	31,937	0.0128	42,985	0.0172	57,616	0.0230	77,203	0.0309	209,742	0.0839
31	LZ	34,136	0.0137	30,495	0.0122	40,160	0.0161	157,528	0.0630	262,318	0.1049
32	LZ	32,138	0.0129	10,802	0.0043	19,077	0.0076	78,361	0.0313	140,377	0.0562
33	LZ	19,996	0.0080	32,703	0.0131	87,147	0.0349	113,186	0.0453	253,031	0.1012
34	LZ	48,159	0.0193	30,452	0.0122	48,581	0.0194	76,943	0.0308	204,134	0.0817
35	LZ	16,765	0.0067	48,245	0.0193	56,550	0.0226	70,737	0.0283	192,296	0.0769
36	LZ	33,788	0.0135	15,217	0.0061	9,075	0.0036			58,080	0.0232
37	LZ	1,397	0.0006							1,397	0.0006
38	LZ	8,853	0.0035	13,361	0.0053					22,215	0.0089
		Min	0.0006		0.0017		0.0034		0.0110		0.0006
		Max	0.0193		0.0275		0.0349		0.0982		0.1492
		Mean	0.0089		0.0109		0.0202		0.0385		0.0567
		SD	0.0046		0.0067		0.0117		0.0242		0.0441
