# Supplementary material for: Genomic Analysis of Latvian Brown Old Type and Latvian Blue Local Dairy Cattle Breeds Using SNP Data
Source: Animals (Basel). 2025 Dec 20;16(1):20. doi: 10.3390/ani16010020 (PMC12784749; doi:10.3390/ani16010020)
Supplement: Supplementary file 1 [file animals-16-00020-s001.zip › Table S4.pdf]

Table S4. FROH by total ROH and by ROH category in BV and LZ breeds' cows.

Cow code	Breed	ROH 1–4 Mb		ROH 4–8 Mb		ROH 8–16 Mb		ROH > 16 Mb		Total	
		Total ROH, KB	FROH	Total ROH, KB	FROH	Total ROH, KB	FROH	Total ROH, KB	FROH	Total ROH, KB	FROH
1	BV	22,203	0.0089	4,423	0.0018	9,001	0.0036			35,627	0.0143
2	BV	50,489	0.0202	48,394	0.0194	26,837	0.0107	136,247	0.0545	261,966	0.1048
3	BV	83,466	0.0334	62,912	0.0252	70,779	0.0283	19,994	0.0080	237,151	0.0949
4	BV	66,516	0.0266	34,913	0.0140	60,164	0.0241			161,593	0.0646
5	BV	32,126	0.0129	31,365	0.0125	29,136	0.0117			92,627	0.0371
6	BV	58,986	0.0236	35,720	0.0143	75,700	0.0303	29,128	0.0117	199,534	0.0798
7	BV	40,955	0.0164	41,208	0.0165	56,261	0.0225	16,163	0.0065	154,586	0.0618
8	BV	49,977	0.0200	4,412	0.0018	9,533	0.0038			63,922	0.0256
9	BV	50,630	0.0203	67,688	0.0271	63,988	0.0256	22,346	0.0089	204,651	0.0819
10	BV	61,822	0.0247	80,047	0.0320	40,544	0.0162			182,414	0.0730
11	BV	74,671	0.0299	108,587	0.0434	72,885	0.0292	40,834	0.0163	296,977	0.1188
12	BV	61,073	0.0244	71,936	0.0288	87,768	0.0351	194,408	0.0778	415,186	0.1661
13	BV	76,189	0.0305	30,219	0.0121	68,462	0.0274			174,870	0.0699
14	BV	60,668	0.0243	97,102	0.0388	53,292	0.0213	77,068	0.0308	288,129	0.1153
15	BV	69,513	0.0278	48,936	0.0196	70,892	0.0284	17,795	0.0071	207,136	0.0829
16	BV	68,183	0.0273	41,068	0.0164	42,430	0.0170	35,162	0.0141	186,844	0.0747
17	BV	47,757	0.0191	61,816	0.0247	58,569	0.0234			168,142	0.0673
18	BV	86,774	0.0347	75,868	0.0303	53,802	0.0215	46,160	0.0185	262,603	0.1050
19	BV	57,523	0.0230	65,416	0.0262	53,200	0.0213	17,003	0.0068	193,141	0.0773
20	BV	74,790	0.0299	32,536	0.0130			34,624	0.0138	141,950	0.0568
21	BV	54,592	0.0218	38,848	0.0155	76,040	0.0304	90,600	0.0362	260,079	0.1040
22	BV	75,559	0.0302	57,889	0.0232	41,647	0.0167			175,096	0.0700
23	BV	57,301	0.0229	47,973	0.0192	28,251	0.0113	24,951	0.0100	158,476	0.0634
24	BV	59,782	0.0239	108,542	0.0434	17,318	0.0069	49,813	0.0199	235,456	0.0942
25	BV	85,535	0.0342	77,267	0.0309	92,596	0.0370	47,862	0.0191	303,260	0.1213

Cow code	Breed	ROH 1–4 Mb		ROH 4–8 Mb		ROH 8–16 Mb		ROH > 16 Mb		Total	
		Total ROH, KB	F_{ROH}	Total ROH, KB	F_{ROH}	Total ROH, KB	F_{ROH}	Total ROH, KB	F_{ROH}	Total ROH, KB	F_{ROH}
26	BV	48,654	0.0195	40,534	0.0162	52,109	0.0208	78,851	0.0315	220,148	0.0881
27	BV	70,875	0.0284	66,592	0.0266	29,599	0.0118			167,066	0.0668
28	BV	54,752	0.0219	37,851	0.0151	111,421	0.0446	22,346	0.0089	226,370	0.0905
29	BV	47,460	0.0190	29,518	0.0118	20,420	0.0082			97,399	0.0390
30	BV	69,175	0.0277	68,667	0.0275	82,352	0.0329	45,845	0.0183	266,039	0.1064
31	BV	63,980	0.0256	67,463	0.0270	67,314	0.0269	145,103	0.0580	343,861	0.1375
32	BV	61,510	0.0246	79,238	0.0317	10,113	0.0040	28,231	0.0113	179,093	0.0716
33	BV	20,079	0.0080	23,056	0.0092	18,099	0.0072	43,304	0.0173	104,538	0.0418
34	BV	42,968	0.0172	14,016	0.0056	20,302	0.0081	35,906	0.0144	113,192	0.0453
35	BV	69,532	0.0278	65,117	0.0260	88,081	0.0352	155,955	0.0624	378,685	0.1515
36	BV	50,059	0.0200	61,538	0.0246	47,209	0.0189	16,367	0.0065	175,173	0.0701
37	BV	56,379	0.0226	53,172	0.0213	92,563	0.0370			202,113	0.0808
38	BV	75,453	0.0302	52,733	0.0211	48,307	0.0193	93,479	0.0374	269,972	0.1080
39	BV	55,156	0.0221	69,318	0.0277	72,263	0.0289	17,576	0.0070	214,313	0.0857
40	BV	75,081	0.0300	40,826	0.0163	60,783	0.0243	38,265	0.0153	214,955	0.0860
41	BV	62,508	0.0250	73,424	0.0294	52,417	0.0210	86,684	0.0347	275,033	0.1100
42	BV	73,153	0.0293	60,725	0.0243	113,572	0.0454			247,449	0.0990
43	BV	61,993	0.0248	36,592	0.0146	63,658	0.0255	43,760	0.0175	206,003	0.0824
44	BV	102,317	0.0409	40,031	0.0160	88,982	0.0356	17,134	0.0069	248,464	0.0994
45	BV	37,584	0.0150	58,520	0.0234	18,874	0.0075	50,318	0.0201	165,295	0.0661
46	BV	85,826	0.0343	68,798	0.0275	59,275	0.0237	60,336	0.0241	274,236	0.1097
47	BV	89,497	0.0358	77,052	0.0308	68,324	0.0273	95,203	0.0381	330,075	0.1320
48	BV	78,575	0.0314	64,975	0.0260	96,986	0.0388			240,536	0.0962
49	BV	79,723	0.0319	60,471	0.0242	48,986	0.0196	103,062	0.0412	292,242	0.1169
50	BV	39,569	0.0158	39,815	0.0159	41,631	0.0167	17,529	0.0070	138,544	0.0554
51	BV	82,907	0.0332	29,746	0.0119	57,167	0.0229	41,073	0.0164	210,894	0.0844
52	BV	63,843	0.0255	40,324	0.0161	52,277	0.0209	16,322	0.0065	172,767	0.0691

Cow code	Breed	ROH 1–4 Mb		ROH 4–8 Mb		ROH 8–16 Mb		ROH > 16 Mb		Total	
		Total ROH, KB	F_{ROH}	Total ROH, KB	F_{ROH}	Total ROH, KB	F_{ROH}	Total ROH, KB	F_{ROH}	Total ROH, KB	F_{ROH}
53	BV	55,746	0.0223	56,445	0.0226	25,981	0.0104	38,248	0.0153	176,421	0.0706
54	BV	50,864	0.0203	36,237	0.0145	73,848	0.0295	60,205	0.0241	221,154	0.0885
55	BV	71,451	0.0286	63,652	0.0255	46,567	0.0186	40,262	0.0161	221,932	0.0888
56	BV	88,088	0.0352	41,648	0.0167	62,030	0.0248	24,004	0.0096	215,771	0.0863
57	BV	67,230	0.0269	63,778	0.0255	40,096	0.0160	37,624	0.0150	208,728	0.0835
58	BV	64,026	0.0256	89,555	0.0358	64,908	0.0260	223,956	0.0896	442,446	0.1770
59	BV	53,264	0.0213	93,161	0.0373	76,682	0.0307	37,907	0.0152	261,014	0.1044
60	BV	63,469	0.0254	59,512	0.0238	9,445	0.0038	93,673	0.0375	226,100	0.0904
61	BV	48,038	0.0192	55,786	0.0223	46,298	0.0185	78,000	0.0312	228,121	0.0912
62	BV	86,803	0.0347	87,535	0.0350	87,862	0.0351	50,257	0.0201	312,457	0.1250
63	BV	39,380	0.0158	81,776	0.0327	26,443	0.0106	19,873	0.0079	167,472	0.0670
64	BV	58,915	0.0236	43,636	0.0175	50,678	0.0203	18,446	0.0074	171,676	0.0687
65	BV	63,642	0.0255	31,370	0.0125	65,814	0.0263	43,264	0.0173	204,090	0.0816
66	BV	61,914	0.0248	34,331	0.0137	51,956	0.0208	129,316	0.0517	277,517	0.1110
67	BV	93,579	0.0374	100,347	0.0401	83,910	0.0336	48,322	0.0193	326,158	0.1305
68	BV	48,942	0.0196	15,659	0.0063	42,305	0.0169			106,905	0.0428
69	BV	33,916	0.0136	30,094	0.0120	57,180	0.0229	26,749	0.0107	147,940	0.0592
70	BV	60,838	0.0243	53,894	0.0216	29,982	0.0120			144,715	0.0579
71	BV	39,116	0.0156	98,099	0.0392	94,254	0.0377	76,711	0.0307	308,181	0.1233
72	BV	71,312	0.0285	47,184	0.0189	54,621	0.0218			173,117	0.0692
73	BV	37,849	0.0151	17,862	0.0071	40,963	0.0164	60,113	0.0240	156,787	0.0627
74	BV	84,486	0.0338	61,130	0.0245	46,595	0.0186	138,527	0.0554	330,737	0.1323
75	BV	77,201	0.0309	59,547	0.0238	42,359	0.0169	111,537	0.0446	290,644	0.1163
76	BV	75,054	0.0300	44,552	0.0178	30,472	0.0122	31,374	0.0125	181,452	0.0726
77	BV	44,285	0.0177	21,880	0.0088	25,901	0.0104	17,598	0.0070	109,665	0.0439
78	BV	64,001	0.0256	34,210	0.0137	80,710	0.0323	40,935	0.0164	219,856	0.0879
79	BV	73,354	0.0293	49,748	0.0199	73,984	0.0296	38,380	0.0154	235,465	0.0942

Cow code	Breed	ROH 1–4 Mb		ROH 4–8 Mb		ROH 8–16 Mb		ROH > 16 Mb		Total	
		Total ROH, KB	F_{ROH}	Total ROH, KB	F_{ROH}	Total ROH, KB	F_{ROH}	Total ROH, KB	F_{ROH}	Total ROH, KB	F_{ROH}
80	BV	23,136	0.0093	28,068	0.0112	12,731	0.0051	25,657	0.0103	89,591	0.0358
81	BV	42,465	0.0170	25,143	0.0101	32,926	0.0132	39,423	0.0158	139,956	0.0560
82	BV	40,368	0.0161	55,462	0.0222	9,067	0.0036	82,734	0.0331	187,630	0.0751
83	BV	76,712	0.0307	56,845	0.0227	51,004	0.0204	73,228	0.0293	257,789	0.1031
84	BV	57,589	0.0230	65,236	0.0261	88,881	0.0356	85,749	0.0343	297,455	0.1190
85	BV	62,365	0.0249	43,880	0.0176	51,688	0.0207			157,933	0.0632
86	BV	58,112	0.0232	56,753	0.0227	67,337	0.0269	72,580	0.0290	254,782	0.1019
87	BV	40,214	0.0161	48,136	0.0193	17,344	0.0069			105,693	0.0423
88	BV	33,968	0.0136	6,817	0.0027					40,785	0.0163
89	BV	63,461	0.0254	60,567	0.0242	50,657	0.0203	16,374	0.0065	191,058	0.0764
90	BV	71,433	0.0286	54,443	0.0218	85,685	0.0343	61,372	0.0245	272,933	0.1092
91	BV	63,921	0.0256	67,087	0.0268	13,569	0.0054	80,711	0.0323	225,288	0.0901
92	BV	65,142	0.0261	65,026	0.0260	140,111	0.0560	55,409	0.0222	325,687	0.1303
93	BV	57,599	0.0230	99,942	0.0400	66,988	0.0268	18,517	0.0074	243,046	0.0972
94	BV	59,426	0.0238	83,456	0.0334	53,027	0.0212	63,552	0.0254	259,460	0.1038
		Min	0.0080		0.0018		0.0036		0.0065		0.0143
		Max	0.0409		0.0434		0.0560		0.0896		0.1770
		Mean	0.0244		0.0216		0.0218		0.0230		0.0858
		SD	0.0066		0.0092		0.0106		0.0170		0.0305

Cow code	Breed	ROH 1–4 Mb		ROH 4–8 Mb		ROH 8–16 Mb		ROH > 16 Mb		Total	
		Total ROH, KB	F_{ROH}	Total ROH, KB	F_{ROH}	Total ROH, KB	F_{ROH}	Total ROH, KB	F_{ROH}	Total ROH, KB	F_{ROH}
95	LZ	35,185	0.0141	59,437	0.0238	72,083	0.0288	95,128	0.0381	261,833	0.1047
96	LZ	50,986	0.0204	30,971	0.0124	82,312	0.0329	52,626	0.0211	216,896	0.0868
97	LZ	18,118	0.0072	7,569	0.0030					25,687	0.0103
98	LZ	19,182	0.0077	6,499	0.0026	19,533	0.0078	58,603	0.0234	103,817	0.0415
99	LZ	34,824	0.0139	23,913	0.0096	26,687	0.0107	121,180	0.0485	206,603	0.0826
100	LZ	40,672	0.0163	15,856	0.0063	10,547	0.0042	157,671	0.0631	224,746	0.0899
101	LZ	47,697	0.0191	69,894	0.0280	100,317	0.0401	177,026	0.0708	394,934	0.1580
102	LZ	48,119	0.0192	36,719	0.0147	66,303	0.0265	165,576	0.0662	316,716	0.1267
103	LZ	44,018	0.0176	35,823	0.0143	89,310	0.0357	62,232	0.0249	231,382	0.0926
104	LZ	56,619	0.0226	52,613	0.0210	128,941	0.0516	357,131	0.1429	595,305	0.2381
105	LZ	18,484	0.0074	4,271	0.0017					22,755	0.0091
106	LZ	42.885	0.0172	21,650	0.0087	34,593	0.0138	227,512	0.0910	326,640	0.1307
107	LZ	59,767	0.0239	35,256	0.0141	84,416	0.0338	99,329	0.0397	278,767	0.1115
108	LZ	30,663	0.0123	32,846	0.0131	31,318	0.0125	99,364	0.0397	194,191	0.0777
109	LZ	19,604	0.0078							19,604	0.0078
110	LZ	16,174	0.0065							16,174	0.0065
111	LZ	34,292	0.0137	27,245	0.0109	14,995	0.0060			76,532	0.0306
112	LZ	70,767	0.0283	38,632	0.0155	64,791	0.0259	77,874	0.0311	252,064	0.1008
113	LZ	42,970	0.0172	23,509	0.0094	73,936	0.0296	184,027	0.0736	324,442	0.1298
114	LZ	42,856	0.0171	28,138	0.0113	96,256	0.0385	93,028	0.0372	260,279	0.1041
115	LZ	41,552	0.0166	55,424	0.0222	71,789	0.0287	228,351	0.0913	397,116	0.1588
116	LZ	47,629	0.0191	51,539	0.0206	102,241	0.0409	228,881	0.0916	430,290	0.1721
117	LZ	30,423	0.0122	34,567	0.0138	72,095	0.0288	57,291	0.0229	194,376	0.0778
118	LZ	50,958	0.0204	25,879	0.0104	8,798	0.0035	103,006	0.0412	188,641	0.0755
119	LZ	61,087	0.0244	48,736	0.0195	47,277	0.0189	132,524	0.0530	289,625	0.1158
120	LZ	26,534	0.0106	22,802	0.0091	51,296	0.0205	31,832	0.0127	132,465	0.0530

Cow code	Breed	ROH 1–4 Mb		ROH 4–8 Mb		ROH 8–16 Mb		ROH > 16 Mb		Total	
		Total ROH, KB	F_{ROH}	Total ROH, KB	F_{ROH}	Total ROH, KB	F_{ROH}	Total ROH, KB	F_{ROH}	Total ROH, KB	F_{ROH}
121	LZ	55,989	0.0224	34,041	0.0136	55,001	0.0220	59,278	0.0237	204,309	0.0817
122	LZ	66,928	0.0268	82,287	0.0329	74,826	0.0299			224,041	0.0896
123	LZ	38,652	0.0155	47,946	0.0192	10,758	0.0043	64,846	0.0259	162,201	0.0649
124	LZ	45,137	0.0181	46,686	0.0187	91,404	0.0366	76,483	0.0306	259,711	0.1039
125	LZ	45,467	0.0182	42,640	0.0171	43,406	0.0174	25,658	0.0103	157,171	0.0629
126	LZ	43,290	0.0173	40,369	0.0161	34,890	0.0140	175,317	0.0701	293,866	0.1175
127	LZ	36,611	0.0146	33,838	0.0135	35,123	0.0140	91,585	0.0366	197,157	0.0789
128	LZ	40,754	0.0163	40,970	0.0164	21,733	0.0087	99,793	0.0399	203,250	0.0813
129	LZ	58,455	0.0234	24,012	0.0096	106,500	0.0426	177,230	0.0709	366,198	0.1465
130	LZ	35,482	0.0142	32,040	0.0128	67,937	0.0272	94,574	0.0378	230,032	0.0920
131	LZ	44,017	0.0176	27,608	0.0110	30,458	0.0122	200,611	0.0802	302,694	0.1211
132	LZ	35,428	0.0142	19,478	0.0078	49,098	0.0196	33,574	0.0134	137,578	0.0550
133	LZ	39,821	0.0159	27,197	0.0109	83,295	0.0333			150,313	0.0601
134	LZ	37,141	0.0149	33,665	0.0135	48,429	0.0194	210,946	0.0844	330,181	0.1321
135	LZ	51,430	0.0206	43,355	0.0173	102,866	0.0411	53,199	0.0213	250,849	0.1003
136	LZ	64,282	0.0257	58,670	0.0235	61,865	0.0247	148,960	0.0596	333,777	0.1335
137	LZ	40,908	0.0164	17,340	0.0069	38,559	0.0154	124,698	0.0499	221,505	0.0886
138	LZ	21,090	0.0084	7,854	0.0031					28,944	0.0116
139	LZ	21,520	0.0086	12,638	0.0051	23,198	0.0093			57,355	0.0229
140	LZ	31,862	0.0127	13,314	0.0053	15,621	0.0062	43,083	0.0172	103,880	0.0416
141	LZ	24,411	0.0098			10,884	0.0044	26,095	0.0104	61,391	0.0246
142	LZ	25,429	0.0102							25,429	0.0102
143	LZ	41,725	0.0167	24,328	0.0097	64,109	0.0256	157,613	0.0630	287,776	0.1151
144	LZ	44,386	0.0178	46,920	0.0188	40,668	0.0163	131,055	0.0524	263,029	0.1052
145	LZ	47,907	0.0192	89,647	0.0359	66,484	0.0266	329,179	0.1317	533,217	0.2133
146	LZ	29,632	0.0119	7,052	0.0028	14,131	0.0057			50,816	0.0203
147	LZ	25,789	0.0103	15,968	0.0064	21,505	0.0086	300,388	0.1202	363,650	0.1455

Cow code	Breed	ROH 1–4 Mb		ROH 4–8 Mb		ROH 8–16 Mb		ROH > 16 Mb		Total	
		Total ROH, KB	F_{ROH}	Total ROH, KB	F_{ROH}	Total ROH, KB	F_{ROH}	Total ROH, KB	F_{ROH}	Total ROH, KB	F_{ROH}
148	LZ	38,477	0.0154	60,297	0.0241	22,693	0.0091	40,193	0.0161	161,659	0.0647
149	LZ	14,894	0.0060							14,894	0.0060
150	LZ	37,194	0.0149	35,479	0.0142	36,557	0.0146	57,118	0.0228	166,348	0.0665
151	LZ	22,980	0.0092	4,226	0.0017	9,105	0.0036			36,312	0.0145
152	LZ	45,857	0.0183	24,769	0.0099	74,650	0.0299	39,588	0.0158	184,863	0.0739
153	LZ	35,166	0.0141	23,214	0.0093	14,396	0.0058	179,986	0.0720	252,762	0.1011
154	LZ	26,275	0.0105	25,986	0.0104	37,383	0.0150	217,325	0.0869	306,969	0.1228
155	LZ	41,658	0.0167	31,104	0.0124	37,466	0.0150	97,979	0.0392	208,208	0.0833
156	LZ	43,758	0.0175	41,074	0.0164	41,525	0.0166	37,939	0.0152	164,296	0.0657
157	LZ	12,875	0.0052					18,292	0.0073	31,168	0.0125
158	LZ	35,007	0.0140	47,571	0.0190	40,120	0.0160	171,782	0.0687	294,480	0.1178
159	LZ	43,376	0.0174	41,170	0.0165	73,162	0.0293	75,512	0.0302	233,220	0.0933
160	LZ	21,318	0.0085	19,746	0.0079	57,516	0.0230	131,640	0.0527	230,220	0.0921
161	LZ	30,254	0.0121							30,254	0.0121
162	LZ	2,993	0.0012							2,993	0.0012
163	LZ	41,857	0.0167	39,150	0.0157	28,003	0.0112	74,333	0.0297	183,343	0.0733
164	LZ	31,480	0.0126	40,308	0.0161	22,196	0.0089			93,984	0.0376
165	LZ	43,890	0.0176	25,377	0.0102	27,589	0.0110	109,593	0.0438	206,450	0.0826
166	LZ	20,568	0.0082	51,813	0.0207	24,480	0.0098	127,693	0.0511	224,553	0.0898
		Min	0.0012		0.0017		0.0035		0.0073		0.0012
		Max	0.0283		0.0359		0.0516		0.1429		0.2381
		Mean	0.0151		0.0136		0.0201		0.0487		0.0823
		SD	0.0054		0.0071		0.0119		0.0310		0.0496
