# Supplementary material for: Genomic Analysis of Latvian Brown Old Type and Latvian Blue Local Dairy Cattle Breeds Using SNP Data
Source: Animals (Basel). 2025 Dec 20;16(1):20. doi: 10.3390/ani16010020 (PMC12784749; doi:10.3390/ani16010020)
Supplement: Supplementary file 1 [file animals-16-00020-s001.zip › Table S5.pdf]

Table S5. First lactation cows' productivity and ROH-based inbreeding (F_{ROH}) in BV and LZ breeds.

Cow code	Breed	F_{ROH}, %	Milk yield, kg in 305 days	Fat content, %	Protein content, %	ECM, kg
1	BV	1.43	5,788	4.77	3.38	6,319.0
2	BV	10.48	4,309	4.4	3.54	4,563.0
3	BV	9.49	5,648	4.39	3.27	5,856.5
11	BV	11.88	4,188	5.57	3.92	5,155.2
80	BV	3.58	6,780	4.88	3.36	7,482.5
81	BV	5.60	4,748	4.24	3.47	4,909.6
82	BV	7.51	4,268	3.92	3.3	4,190.7
83	BV	10.31	4,155	4.46	3.71	4,484.8
84	BV	11.90	3,750	4.98	4.07	4,389.5
85	BV	6.32	4,575	5.16	3.56	5,275.8
86	BV	10.19	2,595	4.8	3.73	2,912.6
87	BV	4.23	4,164	4.33	3.71	4,428.4
88	BV	1.63	4,149	3.78	3.54	4,079.8
89	BV	7.64	5,016	4.31	3.47	5,229.5
90	BV	10.92	3,453	4.5	3.93	3,802.4
12	BV	16.61	3,123	4.79	3.46	3,436.4
13	BV	6.99	4,164	5.17	3.41	4,758.8
14	BV	11.53	3,780	5.12	3.47	4,314.4
15	BV	8.29	4,629	5.14	3.41	5,273.3
16	BV	7.47	4,855	4.66	3.2	5,167.9
17	BV	6.73	4,402	5.37	3.59	5,199.3
18	BV	10.50	3,191	5.04	3.7	3,667.5
19	BV	7.73	3,879	5.46	3.69	4,654.0
20	BV	5.68	3,645	5.02	3.39	4,093.4
21	BV	10.40	3,870	4.9	3.45	4,307.3
22	BV	7.00	4,265	4.81	3.48	4,710.0
23	BV	6.34	2,991	4.87	3.61	3,354.9
24	BV	9.42	3,472	4.91	3.85	3,975.6
25	BV	12.13	2,866	5.55	3.88	3,512.0
26	BV	8.81	3,497	4.69	3.19	3,732.5
27	BV	6.68	4,660	5.19	3.54	5,383.7
28	BV	9.05	4,116	5.87	3.33	5,030.0
29	BV	3.90	4,090	5.55	3.52	4,898.5
30	BV	10.64	3,229	5.7	3.76	3,986.1
31	BV	13.75	4,333	5.41	3.41	5,078.8
32	BV	7.16	2,790	4.67	3.26	2,986.1
33	BV	4.18	4,551	5.09	3.33	5,128.6
34	BV	4.53	3,263	5.39	3.4	3,814.1
35	BV	15.15	4,622	5.49	3.38	5,451.9
36	BV	7.01	4,511	4.77	3.04	4,806.6
37	BV	8.08	4,203	4.98	3.03	4,582.9
38	BV	10.80	5,344	5.47	3.66	6,405.9
39	BV	8.57	3,717	5.48	3.8	4,500.2
40	BV	8.60	4,512	4.95	3.31	5,000.7

Cow code	Breed	F_{ROH}, %	Milk yield, kg in 305 days	Fat content, %	Protein content, %	ECM, kg
41	BV	11.00	3,587	5.17	3.63	4,160.2
42	BV	9.90	3,195	4.92	3.56	3,590.9
43	BV	8.24	2,655	5.21	3.5	3,065.6
44	BV	9.94	2,456	5.61	3.32	2,921.6
45	BV	6.61	2,567	4.76	3.77	2,876.5
46	BV	10.97	2,845	5.19	3.34	3,243.0
47	BV	13.20	2,958	4.71	3.43	3,219.1
49	BV	11.69	3,612	4.08	3.15	3,575.4
50	BV	5.54	3,448	5.36	3.69	4,094.8
51	BV	8.44	3,869	5.07	3.55	4,416.2
52	BV	6.91	3,657	5.52	3.4	4,332.7
53	BV	7.06	3,831	5.5	3.35	4,514.7
55	BV	8.88	3,116	5.38	3.91	3,761.0
56	BV	8.63	3,213	4.77	3.73	3,594.4
57	BV	8.35	3,893	4.86	3.19	4,235.9
58	BV	17.70	3,014	5.59	3.81	3,691.9
59	BV	10.44	3,429	4.91	3.68	3,881.4
60	BV	9.04	2,993	4.42	3.44	3,153.6
61	BV	9.12	3,827	5.73	3.79	4,747.1
62	BV	12.50	3,723	6.44	4.26	5,075.4
64	BV	6.87	4,288	4.66	3.4	4,630.5
65	BV	8.16	2,948	5.4	4.02	3,590.4
4	BV	6.46	4,839	4.08	3.33	4,857.0
5	BV	3.71	4,845	4.19	3.23	4,890.7
6	BV	7.98	5,207	3.87	3.2	5,040.9
7	BV	6.18	5,747	4.5	3.4	6,093.8
8	BV	2.56	6,778	4.42	3.8	7,329.9
9	BV	8.19	2,633	5.3	4.26	3,223.3
10	BV	7.30	4,523	4.76	3.67	5,033.5
91	BV	9.01	4,310	5.13	3.59	4,964.4
92	BV	13.03	4,793	4.2	3.4	4,906.9
93	BV	9.72	4,084	4.35	3.79	4,378.5
94	BV	10.38	5,258	4.29	3.35	5,420.4
66	BV	11.10	4,722	4.49	3.35	4,983.0
98	LZ	4.15	4,876	5.66	3.57	5,924.1
99	LZ	8.26	4,708	4.55	3.32	4,991.8
100	LZ	8.99	4,770	4.82	3.12	5,141.1
130	LZ	9.20	2,115	4.96	3.5	2,377.6
131	LZ	12.11	2,300	5.17	3.53	2,649.8
109	LZ	0.78	2,876	4.2	3.21	2,902.2
156	LZ	6.57	2,966	4.76	3.81	3,332.8
166	LZ	8.98	3,075	3.87	3.27	2,993.5
121	LZ	8.17	3,150	4.06	3.51	3,197.8
125	LZ	6.29	3,182	4.48	3.72	3,444.7
122	LZ	8.96	3,203	4.92	3.44	3,570.3
135	LZ	10.03	3,636	3.83	3.3	3,530.3

Cow code	Breed	F_{ROH}, %	Milk yield, kg in 305 days	Fat content, %	Protein content, %	ECM, kg
163	LZ	7.33	3,761	4.11	3.43	3,817.8
123	LZ	6.49	3,854	5.17	3.24	4,354.0
124	LZ	10.39	3,896	5.0	3.7	4,458.8
120	LZ	5.30	3,897	4.56	3.22	4,106.6
127	LZ	7.89	4,146	4.06	3.37	4,164.1
129	LZ	14.65	4,151	5.31	3.16	4,734.9
153	LZ	10.11	4,243	3.99	3.0	4,104.3
111	LZ	3.06	4,297	4.49	3.33	4,527.9
146	LZ	2.03	4,362	4.3	3.13	4,428.1
113	LZ	12.98	4,385	4.65	3.56	4,783.9
139	LZ	2.29	4,443	4.83	3.56	4,944.8
147	LZ	14.55	4,489	4.42	3.36	4,702.3
145	LZ	21.33	4,542	4.47	3.26	4,750.5
134	LZ	13.21	4,606	3.68	3.05	4,299.0
108	LZ	7.77	4,765	4.48	3.19	4,963.8
110	LZ	0.65	4,797	4.4	3.39	5,024.3
115	LZ	15.88	4,823	5.15	3.81	5,648.9
104	LZ	23.81	4,833	4.76	3.49	5,311.5
154	LZ	12.28	4,860	3.67	3.18	4,578.9
161	LZ	1.21	4,987	4.26	3.27	5,092.0
102	LZ	12.67	4,993	4.3	3.52	5,218.7
141	LZ	2.46	5,133	4.05	3.07	5,030.5
150	LZ	6.65	5,271	4.16	3.1	5,248.6
138	LZ	1.16	5,289	4.48	3.13	5,485.2
144	LZ	10.52	5,336	4.45	3.11	5,506.2
143	LZ	11.51	5,350	4.64	3.36	5,747.7
133	LZ	6.01	5,609	4.56	3.65	6,096.6
107	LZ	11.15	5,624	5	3.63	6,406.1
106	LZ	13.07	5,625	4.99	3.41	6,305.0
140	LZ	4.16	5,653	5.02	3.5	6,396.3
152	LZ	7.39	5,821	3.92	3.16	5,652.8
151	LZ	1.45	6,383	4.38	3.78	6,861.7
114	LZ	10.41	6,474	4.84	3.48	7,173.1
157	LZ	1.25	6,566	5.1	3.98	7,736.3
103	LZ	9.26	6,687	5.65	3.28	7,966.7
164	LZ	3.76	6,685	4.85	3.69	7,523.2
