# Supplementary figures and images for: Genomic Analysis of Latvian Brown Old Type and Latvian Blue Local Dairy Cattle Breeds Using SNP Data
Source: Animals (Basel). 2025 Dec 20;16(1):20. doi: 10.3390/ani16010020 (PMC12784749; doi:10.3390/ani16010020)

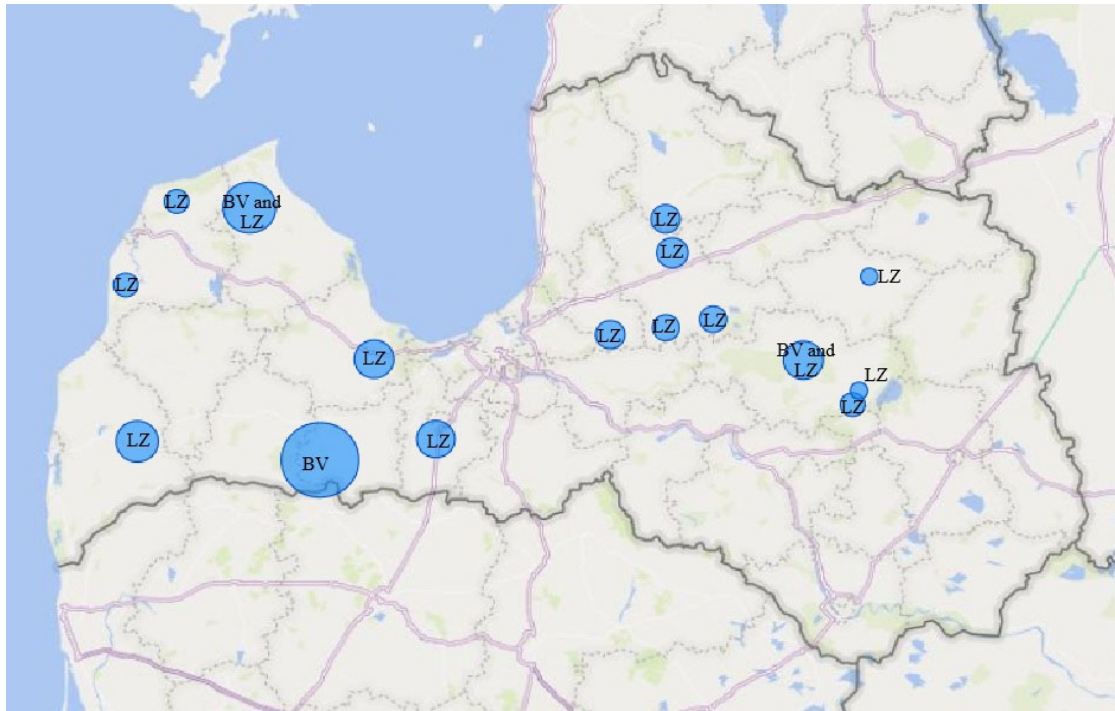

**Figure S1.** Sampling locations for BV and LZ cows.

Supplement: Supplementary file 1 [file animals-16-00020-s001.zip › Figure_S1.pdf]
